# Expression, regulation, and multifaceted molecular and biological functions of sirtuin 6 in the porcine endometrium during early pregnancy

**DOI:** 10.1186/s12964-026-02699-1

**Published:** 2026-02-04

**Authors:** Magdalena Szymanska, Agnieszka Blitek, Kamil Myszczynski

**Affiliations:** InLife ​Institute of Animal Reproduction and Food Research, Polish Academy of Sciences, Trylinskiego 18, Olsztyn, 10-683 Poland

**Keywords:** Sirtuin 6, Endometrium, Implantation, Pregnancy, Pig, Cell cycle, Proliferation, Apoptosis, Adhesion, PGE2

## Abstract

**Background:**

Sirtuin 6 (SIRT6) possesses both deacetylation and mono-ADP-ribosyltransferase activities, affecting diverse biological processes via interaction with cellular substrates. However, the role of SIRT6 in female reproductive functions is largely unknown. This study examined the expression, regulation, and role of SIRT6 in the uterus of early pregnant pigs.

**Methods:**

Endometrial tissue with or without attached trophoblast was collected from gilts on days 10 to 30 of pregnancy to analyze SIRT6 mRNA and protein expression. Endometrial explants and/or luminal epithelial (LE) cells were used to examine the regulation of SIRT6 expression, SIRT6-dependent transcriptomic changes, and the effect of SIRT6 on prostaglandin E2 (PGE2) synthesis, apoptosis, proliferation, cell cycle progression, and cell adhesion.

**Results:**

SIRT6 is expressed at the utero-trophoblast interface during early pregnancy in pigs. RNA sequencing of peri-implantation endometrium after SIRT6 activation with UBCS039 identified 788 up- and 756 down-regulated genes (adjusted *p*-value < 0.05 and log2 fold change > 0.58) that significantly enriched functions attributed to metabolic processes, aminoacyl-tRNA biosynthesis, intracellular protein transport, immune response, apoptotic and cytokine-mediated signaling pathways, cell proliferation and adhesion, and extracellular matrix organization. Many genes were associated with the metabolism of nutrients, steroids, and PGE2, as well as with mitochondrial activity and cell cycle progression. Consistent with elevated expression of genes encoding PG-metabolizing enzymes (*PTGR1*, *AKR1C1*) in the UBCS039-treated endometrial samples, the concentration of PGE2 in culture media was diminished by 90% as compared with the non-treated control (*p* < 0.05). Moreover, the activation of SIRT6 promoted LE cell proliferation, whereas the SIRT6 inhibitor diminished the number of viable cells (*p* < 0.01). In support, UBCS039 accelerated cell cycle progression through the G2/M phase by reducing the levels of cyclin A2 (*p* < 0.05) and B1 (*p* = 0.07). In turn, UBCS039 inhibited cell adhesion (*p* < 0.05).

**Conclusions:**

These results suggest that SIRT6, present at the maternal-conceptus interface in pigs, may modulate endometrial gene expression and support uterine function to maintain pregnancy. Given that implantation failure is a major cause of early embryonic loss in pigs, SIRT6 could be considered a novel target for developing strategies to improve survival of the early conceptuses.

**Supplementary Information:**

The online version contains supplementary material available at 10.1186/s12964-026-02699-1.

## Background

The incidence of prenatal mortality in mammals is substantial, often ranging from 20 to 40% [1], depending on species, litter size, and the stage of pregnancy. In pigs, litter-bearing animals, most embryonic losses occur during the first 30 days of pregnancy, which could be in part due to aberrant endometrial preparation for conceptus implantation. In this species, the endometrium under the control of ovarian progesterone (P4) and diverse embryonic signals such as estrogens (mainly estradiol 17β; E2) and inflammatory mediators remodels extensively in both structure and function to accommodate rapid conceptus development and survival [2–4]. These changes are orchestrated by numerous genes, as reflected in the porcine endometrial transcriptome [5–9]. Particularly, a global gene profiling comparing endometrial tissue between three stages of early pregnancy, i.e. period of maternal recognition of pregnancy (day 12), implantation (day 18), and early placentation (days 24–25), identified a huge number of differentially expressed genes (DEGs). Most of them were associated with biological processes indispensable for promoting implantation, most notably cell proliferation, cell adhesion, pro- and anti-apoptotic functions, immune response, cytokine–cytokine receptor interactions, proteolysis, transport of nutrients, and metabolism [7, 8]. Recently, it has become clear that, to grasp the molecular aspects of implantation failure fully, we need to understand the precise regulation of gene expression, which largely relies on epigenetic processes. The importance of epigenetic regulation—mainly DNA methylation—has been reported in the porcine endometrium in response to nutritional restriction [10] and electromagnetic field exposure [11]. Much less is known about enzymes with deacetylase activity, which may epigenetically coordinate spatio-temporal gene expression in the endometrium at the time of implantation in pigs.

Sirtuin 6 (SIRT6) is a member of a highly conserved sirtuin family of nicotinamide adenine dinucleotide (NAD +)-dependent protein deacetylases and mono-adenosine diphosphate (ADP) ribosyltransferases [12]. SIRT6 is primarily a chromatin-associated nuclear protein, but it can also diffuse to the cytoplasm [13–15]. Through its enzymatic activities, SIRT6 modifies gene expression by targeting histones and other cellular proteins, and affects a wide range of biological processes, including glucose metabolism, fatty acid oxidation, DNA damage repair, inflammation, apoptosis, cell cycle, proliferation, and mitochondrial biology [13, 16–18]. However, the role of SIRT6 in reproductive functions is largely unknown, with only a few reports demonstrating its relevance for maintaining the ovarian reserve [14], apoptosis of follicular cells [19], oocyte meiotic maturation [20–22], and early embryo development [21, 23] (in mice, rats, and pigs). Recently, SIRT6 was found to be expressed in the human uterus [15, 24], but studies exploring its role in this tissue are limited and focused on endometrial cancer, with SIRT6 recognized as a putative tumor suppressor [25]. Of interest, a few reports suggested a possible role of a better-known sirtuin, SIRT1, in the events related to early pregnancy, including cytoskeletal reorganization of human endometrial epithelium [26] and the proliferation and migration of porcine luminal epithelial (LE) cells [27], which occur during the later stages of implantation as the uterine–placental interface prepares for folding to increase the surface area available for maternal–fetal exchanges [3]. Until now, however, no data have been available on the expression and function of SIRT6 in the uterus of animal species, including the pig. Given that SIRT6 acts as both “eraser” and “writer” of epigenetic marks by removing an acetyl group or adding a mono-ADP-ribosylation mark, regulating many cellular functions, we hypothesize that this enzyme may affect the preparation of the porcine endometrium for implantation, manifested by changes in gene expression. To verify this hypothesis, we first determined the mRNA and protein expression of SIRT6 in the endometrium during early pregnancy (days 10 to 30) in pigs. Then, we used porcine endometrial explants and/or LE cells to (1) examine the possible regulation of SIRT6 expression by conceptus products and P4; (2) identify SIRT6-induced transcriptomic changes in the endometrium at the time of implantation; (3) determine the effect of SIRT6 on prostaglandin E2 (PGE2) synthesis, apoptotic markers, proliferation, cell cycle progression, and cell adhesion. The enzymatic activity of SIRT6 was manipulated using the synthetic specific activator UBCS039 and the selective inhibitor OSS_128167.

## Methods

### Animals and sample collection

To analyze endometrial SIRT6 expression (ex vivo study), 30 crossbred gilts (Polish Landrace x Duroc) from one commercial herd were used. Gilts were bred 12 and 24 h after detection of the third estrus. The day of the second breeding was considered the first day of pregnancy. Gilts were slaughtered on days 10 (pre-implantation period), 12 (maternal recognition of pregnancy), 15 (initial conceptus adhesion), 20 (attachment to the endometrium for formal implantation), and 30 (initiation of folding for early placentation [28]) of pregnancy (*n* = 6 per day in each group). Days of pregnancy were confirmed based on the size and morphology of conceptuses, as described previously [29]. Endometrial tissue was snap-frozen in liquid nitrogen and stored at − 80 °C until further analysis. For immunostaining, cross-sections of the uterus or uterus with attached conceptus trophoblasts were fixed in 4% paraformaldehyde (PFA) in 0.1 M PBS (pH 7.4).

For in vitro experiments, 5 gilts from one commercial herd that exhibited two estrous cycles of normal length were artificially inseminated 24 and 48 h after detection of the third estrus and slaughtered on days 15–16 of pregnancy to obtain conceptuses (Experiments 1 and 2) and endometrial explants (Experiments 1, 3, and 4) for incubation. Additionally, uteri collected from gilts on days 11–12 of the estrous cycle, obtained from a local slaughterhouse, served for the isolation of LE cells (*n* = 6; Experiments 2 and 5 to 8).

### Incubation of conceptuses

Days 15–16 conceptuses were collected from uteri by flushing of each uterine horn with Dulbecco's Modified Eagle Medium/Nutrient Mixture F-12 (DMEM/F-12; Sigma-Aldrich, St. Luis, MO, USA) containing 0.1% of bovine serum albumin (BSA; Millipore, Kankakee, IL, USA) and antibiotics (100 IU/mL penicillin and 100 μg/mL streptomycin; Sigma-Aldrich). Conceptuses were placed in culture flasks and incubated in DMEM/F-12 supplemented with antibiotics for 24 h at 37 °C in a humidified atmosphere of 95% air and 5% CO_2_ with gentle shaking. After incubation, media from all conceptuses obtained from each gilt were pooled together and centrifuged at 500 × g for 5 min. Basal medium without any contact with conceptuses was also prepared. Media were stored at − 80 °C, and used in Experiments 1 and 2 as conceptus-exposed medium (CEM) and CEM control.

### Isolation of luminal epithelial cells of the endometrium

LE cells of the porcine endometrium were enzymatically isolated as previously described [30]. Briefly, endometrial tissue was digested with 0.3% (wt/vol) dispase (Sigma-Aldrich) in Hank’s balanced salt solution (pH 7.4; Sigma-Aldrich) at 37 °C for 50 min with gentle shaking. Isolated cell suspension was pelleted by centrifugation at 200 × g for 10 min at 8 °C. Red blood cells were lysed with the Red Blood Cell Lysing Buffer (Sigma-Aldrich). LE cells were then washed with Medium 199 (Sigma-Aldrich) supplemented with 5% newborn calf serum (NCS; Sigma-Aldrich) and antibiotics, filtered through a cell strainer with 100 μm nylon mesh (Corning, NY, USA), and counted in a hemocytometer. The cell viability was higher than 90% as assessed by 0.5% (wt/vol) trypan blue dye exclusion. LE cells were used in Experiments 2 and 5 to 8.

### Experiment 1: effect of conceptus products on SIRT6 expression in endometrial explants

Endometrial explants (150–200 mg per vial) were placed in glass vials containing the control medium (DMEM/F-12 with 0.1% BSA and antibiotics) and pre-incubated for 2 h. Then, the medium was removed, and endometrial strips were treated with CEM control or CEM for 24 h. Moreover, to study the effect of selected conceptus products on SIRT6 expression, endometrial explants were treated with the control medium or the control medium containing E2 (0.01 and 0.1 µM [Sigma-Aldrich]), PGE2 (1 and 10 µM [Sigma-Aldrich]), interferon γ (IFNγ; 10 and 50 ng/mL [BioSource International Inc, Camarillo, CA, USA]) or interleukin 1β (IL1β; 10 and 50 ng/mL [Sigma-Aldrich]). After 24 h, endometrial strips were washed with PBS, snap-frozen in liquid nitrogen, and stored at − 80 °C for further use. For all treatments, endometrial tissue obtained from five different gilts was used.

### Experiment 2: effect of conceptus products and progesterone on SIRT6 expression in LE cells

LE cells were seeded in collagen-coated six-well plates (Corning, BioCoat Collagen I Cellware, Kennebunk, ME, USA) at a density of 1 × 10^6^ cells/well in Medium 199 containing 10% NCS and antibiotics. Cells were cultured for 48 h, when monolayers were assessed to be 90% confluent. Afterwards, cells were incubated with CEM control or CEM for 24 h. Moreover, cells were treated for 24 h with the control medium (Medium 199 with 0.1% of BSA and antibiotics) or the control medium containing E2 (0.01 and 0.1 µM), PGE2 (1 and 10 µM), IFNγ (10 and 50 ng/mL), IL1β (10 and 50 ng/mL), P4 (0.1 µM; Sigma-Aldrich) or the combination of E2 (0.1 µM) and P4. Then, cells were washed with PBS, lysed with TRI Reagent (Molecular Research Center, Cincinnati, OH, USA) for RNA extraction or RIPA buffer (50 mM Tris–HCl, pH 7.4; containing 150 mM NaCl, 1% Triton X-100, 1 mM EDTA, and 10 µL/mL Protease inhibitor cocktail [Sigma-Aldrich]) for protein extraction. For all treatments, cells isolated from four (CEM effect) or six (conceptus products and P4 effects) different gilts were used.

### Experiment 3: the effect of SIRT6 activation on endometrial transcriptome

Tissue explants were prepared as described in Experiment 1. Following the pre-incubation step, endometrial strips were treated for 24 h with the control medium or the control medium containing a specific activator of SIRT6, UBCS039 (100 µM; TargetMol, Boston, MA, USA). Then, tissue was washed with PBS, snap-frozen in liquid nitrogen, and stored at − 80 °C for further RNA-sequencing (RNA-seq) and qPCR analyses. For this experiment, endometrial tissue obtained from three different gilts was used.

### Experiment 4: the effect of SIRT6 on the mRNA expression in endometrial explants and the PGE2 release into culture medium

Tissue explants were prepared as described in Experiment 1. Following the pre-incubation step, endometrial strips were incubated with the control medium or the control medium containing specific activator (UBCS039; 75 and 100 µM) or selective inhibitor (OSS_128167; 100 and 125 µM [TargetMol]) of SIRT6. Additionally, tissue was treated with arachidonic acid, ARA (200 µM; Sigma-Aldrich), which was used as a positive control for the PGE2 synthesis. After 24 h, endometrial strips were washed with PBS, snap-frozen in liquid nitrogen, and stored at –80 °C for further qPCR analysis. Culture media were collected for enzyme immunoassay (EIA) of PGE2. Endometrial explants obtained from five (for UBCS039 and OSS_128167 effects) or four (for ARA effect) different gilts were used.

### Experiment 5: the effect of SIRT6 on PGE2 release by LE cells

LE cells were plated as described in Experiment 2. After reaching 90% confluence, cells were treated with the control medium (Medium 199 supplemented with 0.1% BSA and antibiotics) or the control medium containing UBCS039 (100 µM). After 6 and 24 h, culture media were collected for EIA of PGE2. For this experiment, cells isolated from four different gilts were used.

### Experiment 6: the effect of SIRT6 on the proliferation of LE cells

LE cells were seeded in collagen-coated 96-well plates (Corning) at a density of 1 × 10^4^ cells per well in Medium 199 containing 10% NCS and antibiotics. After reaching 60% confluency, cells were treated with the control medium (Medium 199 supplemented with 0.1% BSA and antibiotics) or the control medium containing SIRT6 activator (UBCS039) or inhibitor (OSS_128167) at concentrations of 50, 75, 100, 125 µM each. As a positive control for cell proliferation, 20% NCS was used. After 24 and 48 h, CellTiter 96 Aqueous One Solution Reagent (Promega, Madison, WI, USA) was added into each well (20 μL per well) and the absorbance was measured at 490 nm wavelength. All treatments were performed in triplicate, using cells isolated from four different gilts.

### Experiment 7: effect of SIRT6 activation on cell cycle progression

LE cells were plated and treated as described in Experiment 5. The treatment was performed in duplicate using cells isolated from four different gilts, and at the end of the experiment, cells from duplicate wells were pooled. Subsequently, 1 × 10^6^ cells were fixed in 1% (vol/vol) formaldehyde (Thermo Fisher Scientific, Waltham, MA, USA) in Medium 199 for 10 min and quenched by 2.5 M glycine for 5 min. Cells were washed in PBS, centrifuged (800 × g, 7 min, 8 °C), and suspended in 1 mL of ice-cold PBS. Then, 1 μL of FxCycle Violet stain (Invitrogen by Thermo Fisher Scientific) was added to each sample. Samples were incubated for 30 min at room temperature, protected from light. Flow cytometric analysis of DNA content distribution was performed at 375 nm excitation with a 450/40 bandpass filter.

To examine the effect of SIRT6 on the expression of cell-cycle-related proteins, LE cells were plated and treated as described in Experiment 5. After 6 and/or 24 h of the treatment, cells were washed with PBS and lysed with RIPA buffer supplemented with 10 μL/mL Protease Inhibitor Cocktail and 20 µL/mL Phosphatase Inhibitor Cocktail 1 (Sigma Aldrich) for protein extraction. For this experiment, cells isolated from three (for cyclins’ analysis) and four (for cyclin-dependent kinases [CDKs] analysis) different gilts were used.

### Experiment 8: the effect of SIRT6 on the adhesive properties of LE cells

LE cells were suspended at a concentration of 1.5 × 10^5^ cells/mL in control medium (Medium 199 supplemented with 1% NCS and antibiotics) or the control medium containing UBCS039 (100 µM). Then, 200 μL (3 × 10^4^ cells) of each cell suspension was transferred into wells of Millicoat Cell Adhesion Strips coated with human fibronectin (Millipore, Billerica, MA, USA) and incubated at 37 °C for 24 h. After the treatment, strips were washed with PBS containing Ca^2+^/Mg^2+^, and cells were fixed with 2% PFA in PBS for 5 min at room temperature. After washing, 100 μL of 0.2% crystal violet in 10% ethanol was added to each well and incubated for 5 min. Then, the plate was washed three times with PBS. Finally, 100 μL of a solubilization buffer (0.1 M NaH_2_PO_4_, pH 4.5, mixed 1:1 with 50% ethanol) was added, and the plate was incubated and gently shaken at room temperature for 5 min. The absorbance was determined colorimetrically at a 550 nm wavelength. For this experiment, cells isolated from three different gilts were used.

### Total RNA extraction, qPCR analysis, and RNA sequencing

Total RNA was extracted from endometrial tissue using a Total RNA Prep Plus kit (A&A Biotechnology, Gdansk, Poland) and from endometrial explants and LE cells using a TRI Reagent and RNeasy Mini Kit (Qiagen, Valencia, CA, USA), following the protocols supplied. For qPCR analysis, samples were treated with DNase I (Sigma-Aldrich) and reverse transcribed using a High-Capacity cDNA Reverse Transcription Kit (Applied Biosystems by Thermo Fisher Scientific). Relative gene expression was assessed using TaqMan Gene Expression Assays (Additional file 1: Table S1), Taq-Man Universal Master Mix II (Applied Biosystems), and an ABI Viia7 Sequence Detection System (Life Technologies Inc., Carlsbad, CA, USA), as described earlier [31]. The qPCR reaction was performed in duplicates using 15 ng of complementary DNA as a template. To test for genomic DNA contamination, the control reactions in the absence of reverse transcriptase were performed. No template control with nuclease-free water was conducted to check for possible reagent contamination. The expression values were calculated using the PCR Miner algorithm [32], normalized to the geometric mean of *GAPDH* and *HPRT1*, and presented as relative expression (arbitrary units).

For RNA-seq (endometrial explants from Experiment 3), sample RNA integrity numbers were evaluated by Agilent Bioanalyzer 2100 (Agilent Technology, USA). The cDNA libraries (poly A enrichment) were prepared and sequenced at Novogene (Novogene, Cambridge, UK). The transcriptome high-throughput sequencing was accomplished on the Illumina platform. To validate the accuracy of the RNA-seq results, eleven randomly selected DEGs were analyzed using qPCR. The names of the tested genes and the ID numbers of TaqMan assays are listed in Additional file 1: Table S1. Pearson correlation of RNA-seq and qPCR results using log2 fold change (log2FC) was calculated.

### Immunofluorescent staining of LE cells

For immunofluorescent staining, cells were cultured in Cell Imaging Coverglasses (Eppendorf, Hamburg, Germany). After reaching confluence, cells were fixed in 4% PFA in PBS (pH 7.4), blocked with PBS containing 1% BSA and 10% donkey normal serum (Jackson ImmunoResearch, West Grove, PA, USA) for 1 h at room temperature. After that, an overnight incubation with monoclonal rabbit anti-SIRT6 antibody (1:20; Abcam, Cambridge, UK) was performed at 4 °C. The slides were subsequently treated with CY3-conjugated donkey anti-rabbit IgG (1:1000; Jackson ImmunoResearch, West Grove, PA, USA). Negative control staining was accomplished by replacing the primary antibody with rabbit IgG negative control (0.1 μg/mL; Vector Laboratories, Inc., Burlingame, CA, USA). Actin filaments were stained with CytoPainter PhalloidiniFluor 488 Reagent (Abcam) according to the manufacturer’s protocol. At the end of incubation, coverslips were rinsed three times with PBS and mounted on slides with VECTASHIELD Mounting Medium containing 4’,6-diamidino-2-phenylindole (DAPI; Vector Laboratories), to visualize cell nuclei. Fluorescent images of the stained cells were captured using a Zeiss microscope (Axio Imager Z1).

### Immunohistochemical staining of the endometrium

The fixed tissues were embedded in paraffin and sectioned at 5 μm. Antigen retrieval was accomplished by microwaving in EPITOPE Retrieval Solution (pH 9.0; Leica Biosystems, Nussloch, Germany) for 15 min. Blocking of non-specific binding sites was performed with Fish Serum Blocking Buffer (Thermo Scientific) for 60 min at room temperature. The overnight incubation with polyclonal rabbit anti-SIRT6 antibody (1:50; Cell Signaling Technology, Danvers, MA, USA) was accomplished at 4 °C in a humidified chamber. Normal rabbit IgG (0.1 μg/mL) was substituted for the primary antibody as a negative control. Tissue sections were washed with TBS (0.05 M TrisBase pH = 7.4, 0.9% NaCl) three times and then incubated with biotinylated goat anti–rabbit IgG (H + L) (1:200; Vector Laboratories) for 30 min at room temperature. Following washes with TBS, avidin–biotin-peroxidase complex (Vectastain Elite ABC Reagent, Vector Laboratories) was added for 30 min. 3,3′-diaminobenzidine (DAB; Sigma-Aldrich) was used as the chromogen. Sections were dehydrated in ethanol, cleared with xylene, mounted in DPX (Sigma-Aldrich), and photographed using a Zeiss microscope (Axio Imager Z1).

### EIA of PGE2

The concentrations of PGE2 in the incubation media were determined using a direct EIA method [33]. An anti-PGE2 antibody (Sigma-Aldrich) developed in rabbits was used at a dilution of 1:200. The sensitivity of the assay was 0.19 ng/mL. The mean intra- and inter-assay coefficients of variation were 2.8 and 7.4%, respectively.

### Protein extraction and western blot analysis

All samples were homogenized using an ice-cold RIPA buffer. Tissue homogenates were then centrifuged for 10 min at 800 × g at 4 °C, and the resulting supernatants were used as the whole-endometrial lysates for Western blot analyses of total SIRT6 protein content. LE cell lysates were kept on ice for 30 min, centrifuged for 10 min at 18,000 × g at 4 °C, and the supernatant was used for the Western blot procedure. Additionally, the nuclear fraction of endometrial tissue from an ex vivo study was prepared. Briefly, tissue was homogenized first in a lysis buffer A (10 mM HEPES, 1.5 mM MgCl2, 10 mM KCl, 0.5 mM DTT, 0.05% IGEPAL, pH 7.9) containing 10 μL/mL Protease inhibitor cocktail and centrifuged for 10 min at 800 × g at 4 °C. Subsequently, supernatants were discarded, and precipitates were lysed in buffer B (5 mM HEPES, 1.5 mM MgCl2, 0.2 mM EDTA, 26% glycerol (v/v), pH 7.9) containing 10 μL/mL Protease Inhibitor Cocktail, and centrifuged for an additional 10 min at 24,000 × g at 4 °C. The resulting supernatants were used as the nuclear fractions for Western blot analysis. Total protein concentrations were determined using the Bradford method [34].

Samples containing equal amounts of total protein were dissolved in SDS gel-loading buffer (50 mM Tris–HCl, pH 6.8; 4% SDS, 20% glycerol, and 2% β-mercaptoethanol), heated to 95 °C for 5 min, and separated on SDS-PAGE or TGX Stain-Free gels (Bio-Rad, Hercules, CA, USA). Separated proteins were electroblotted onto PVDF membrane in transfer buffer (20 mM Tris–HCl, pH 8.2; 150 mM glycine, 20% methanol). Before the transfer, the TGX gels were activated to obtain the total content of loaded protein, according to the manufacturer’s instructions. After 1.5 h of blocking with 5% nonfat dry milk in TBS-T (Tris-buffered saline, containing 0.1% Tween-20), the membranes were incubated overnight at 4 °C with the primary antibodies listed in Additional file 1: Table S2. After washing, the membranes were incubated for 1 h with the anti-rabbit IgG-alkaline phosphatase antibody (1:20,000; Sigma-Aldrich). Immune complexes were visualized by the chemiluminescence procedure using the WESTAR ECL 2.0 kit (Cyanagen, Bologna, Italy). Images were captured in the ChemiDoc™ Touch Imaging System (Bio-Rad, Hercules, CA, USA) and quantified using Image Lab 6 software (Bio-Rad). β-actin or the total protein content in each equivalent lane was used as an internal control for protein loading.

### Bioinformatics analyses

The quality of the generated 2 × 150 bp paired-end raw reads was first quality-checked and cleaned up using FastQC [35] and Trimmomatic [36]. Principal Component Analysis (PCA) for data quality control was performed. FastQC box plots of quality scores per reading position, and Phred score > 30 were also performed. Samples clustering based on a distance matrix created from rlog-transformed gene expression values was done. Then, the trimmed reads were mapped to the *Sus scrofa* genome (Sscrofa11.1) using the STAR mapper [37]. The mapped reads were counted with featureCounts 2.0.3. To compare transcriptomic profiles across samples, PCA was conducted on rlog-transformed count values. To find DEG across groups of samples, DESeq2 [38] was used with an absolute value of log2FC ≥ 0.58 and adjusted *p*-value (padj) < 0.05 as a cutoff. The topGO package [39] was used to test the statistical enrichment of DEG in gene ontology (GO) terms. Only GO terms with a *p*-value < 0.05 were considered significantly enriched. The results of the analysis were visualized using the R package (R Core Team 2021). Search Tool for the Retrieval Interacting Genes (STRING) database was used to construct the network of DEGs and proteins [40].

### Statistical analyses

Statistical analyses were performed using GraphPad PRISM v. 9.3.1 (GraphPad Software, Inc., San Diego, CA, USA). To analyze (1) the expression of SIRT6 in the endometrium of pregnant gilts, and (2) the effects of UBCS039 and OSS_128167 on PGE2 release, *PTGES* mRNA expression, and apoptotic gene expression, one-way ANOVA followed by Tukey's post-hoc test was used. To examine the effect of (3) conceptus products and P4 on SIRT6 expression in endometrial explants and/or LE cells, and (4) UBCS039 and OSS_128167 on cell proliferation, one-way ANOVA followed by Dunnett’s multiple comparison test was used. Student’s t-test was used to study the effect of (5) CEM on SIRT6 expression in endometrial strips and LE cells, (6) ARA on PGE2 release, (7) NCS on cell proliferation, and (8) UBCS039 on phospho-CDK1/2/3 protein level and adhesive properties of cells. To test the effect of (9) UBCS039 on cell cycle distribution and cyclin protein expression, two-way ANOVA followed by Bonferroni’s post hoc test was performed. The Pearson correlation coefficient (r) was used to assess the association between the RNA-seq and qPCR data based on the log2FC of mean expression values. Logarithmic transformation of data was performed for a non-normal distribution assessed using the Shapiro–Wilk test. All numerical data were presented as means ± SEM, and the statistical difference was defined at p < 0.05 with a tendency at *p* = 0.07.

## Results

### SIRT6 is present at the maternal-conceptus interface during early pregnancy

**Fig. 1:**
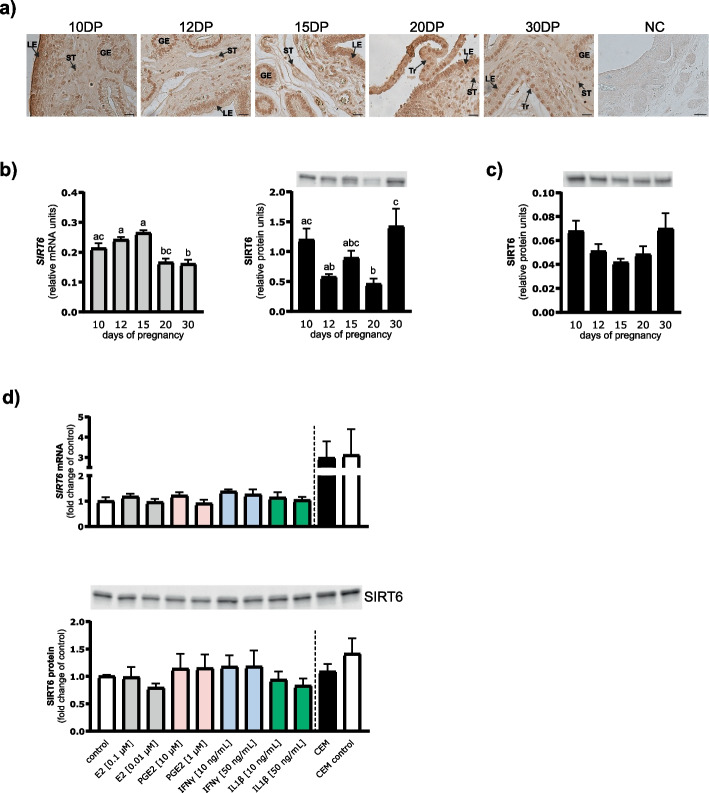
**a-c** Localization and expression of SIRT6 at the maternal-conceptus interface during early pregnancy. **a** Immunohistochemical localization of SIRT6 protein in the endometrium of early pregnant gilts (days 10 to 30) and conceptus trophoblast from days 20 and 30 of pregnancy. Positive immunoreaction was observed in luminal (LE) and glandular (GE) epithelium, stromal (ST) cells of endometrium, as well as in the trophoblast (Tr). Arrows indicate nuclear staining. NC – negative control; DP – day of pregnancy; Scale bars, 20 µm. **b** Expression of SIRT6 mRNA and protein in the endometrium of early pregnant pigs. **c** Expression of SIRT6 protein in the nuclear fraction of the endometrium. **d** Effect of conceptus-derived factors on SIRT6 mRNA and protein expression in endometrial explants. Endometrial slices were treated with estradiol (E2; 0.01 and 0.1 µM), prostaglandin E2 (PGE2; 1 and 10 µM), interferon γ (IFNγ; 10 and 50 ng/mL), interleukin 1β (IL1β; 10 and 50 ng/mL), or conceptus-exposed medium (CEM) for 24 h. Values from qPCR were normalized to the geometric mean of *GAPDH* and *HPRT1* gene expression. Values from densitometric analyses of blots were normalized to total protein content using TGX Stain-Free gel technology. All blots are included in Additional File 3: Fig. S4-S6. **b** and **c** Data are expressed as the mean ± SEM (*n* = 6 per day in each group). Various letters indicate a significant difference between groups (*p* < 0.05). **d** Data are expressed as the mean ± SEM from 5 experiments and presented as a fold change of control values

First, we confirmed that SIRT6 is present at the maternal-conceptus interface during early pregnancy. As demonstrated in Fig. 1a, strong nuclear staining of SIRT6 was observed in luminal and glandular epithelium and stroma of the pig endometrium between days 10 and 30 of pregnancy. Moreover, elongated Day 20 and Day 30 conceptuses showed intense immunoreaction for SIRT6 in the nuclei of trophoblasts. Negative IgG control staining showed no specific immunoreaction for SIRT6.

### SIRT6 mRNA and protein expression in the endometrium of early pregnant gilts

The profile of SIRT6 mRNA and protein expression in the whole endometrial lysates varied during the studied period of early pregnancy (*p* < 0.0001 and *p* = 0.002, respectively; Fig. 1b). Decreased expression of *SIRT6* transcript was detected on day 20 compared with days 12 (*p* = 0.002) and 15 (*p* = 0.0001), and on day 30 compared with days 10 (*p* = 0.047), 12 (*p* = 0.001), and 15 (*p* < 0.0001) of pregnancy. In the total tissue lysates, the lowest level of SIRT6 protein was observed on day 20, compared with day 10 of pregnancy (*p* = 0.039). Thereafter, a marked increase in SIRT6 protein abundance was observed on day 30 compared to days 12 and 20 (*p* = 0.01 and *p* = 0.0045, respectively) of gestation. The content of endometrial SIRT6 protein in the nuclear fraction did not change significantly during the examined period of pregnancy (*p* = 0.08; Fig. 1c).

### Experiment 1: Effect of conceptus products on SIRT6 expression in endometrial explants

The effects of potential conceptus-derived factors on SIRT6 expression in cultured endometrial slices were then assessed (Fig. 1d). None of the selected conceptus products, such as E2, PGE2, IFNγ, or IL1β, affected SIRT6 mRNA and protein abundance in endometrial slices. Culture of endometrial strips with CEM also did not affect SIRT6 expression.

### Experiment 2: SIRT6 expression and its regulation in cultured LE cells

**Fig. 2:**
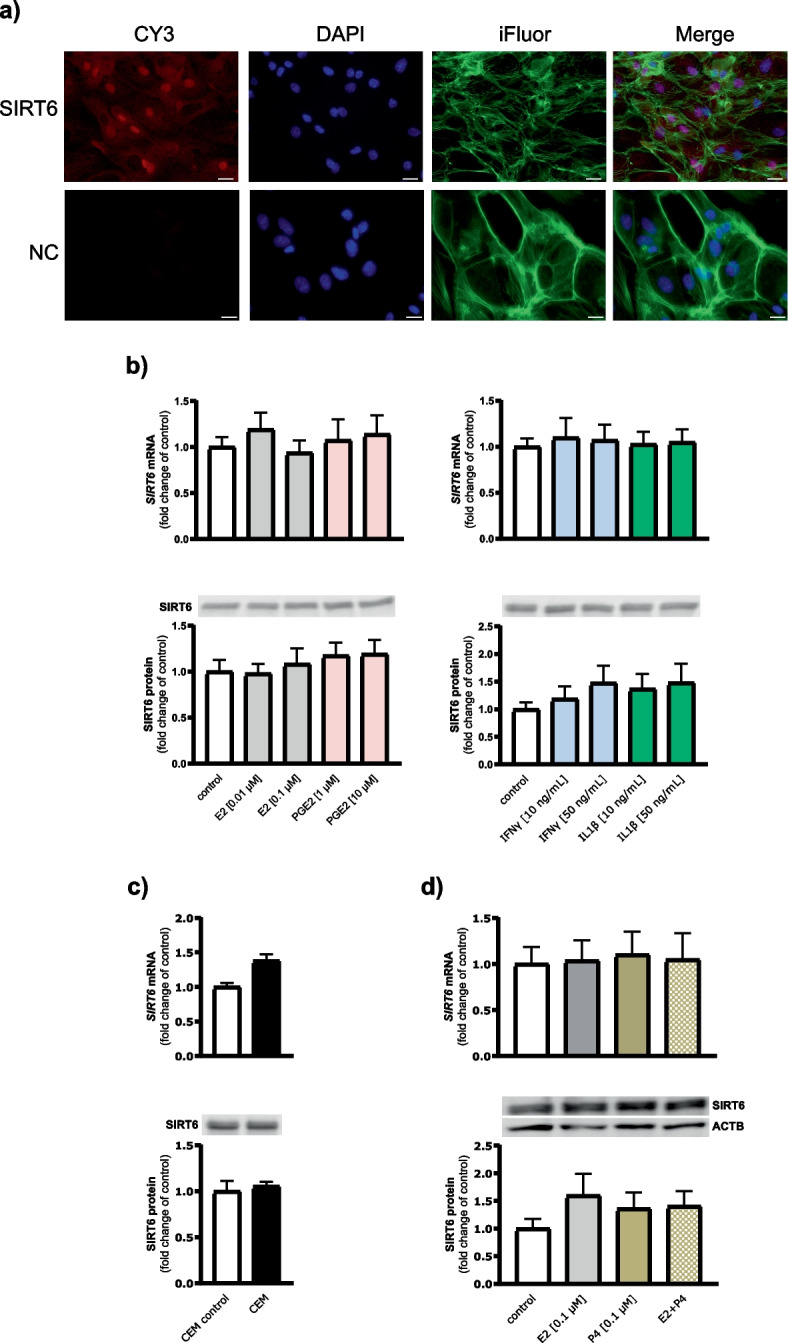
Localization and regulation of SIRT6 expression in luminal epithelial (LE) cells of the porcine endometrium. **a** Representative images showing SIRT6 protein localization in LE cells. Cells were counterstained with diamidino-2-phenylindole (DAPI) and CytoPainter Phalloidin-iFluor 488 Reagent (iFluor) to visualize nuclei and actin filaments, respectively. NC – negative control; scale bars, 20 µm. Cells were treated with (**b**) estradiol (E2; 0.01 and 0.1 µM), prostaglandin E2 (PGE2; 1 and 10 µM), interferon γ (IFNγ; 10 and 50 ng/mL), interleukin 1β (IL1β; 10 and 50 ng/mL), (**c**) conceptus-exposed medium (CEM), (**d**) E2 (0.1 µM), progesterone (P4; 0.1 µM), or the combination of E2 and P4 for 24 h. Values from qPCR were normalized to the geometric mean of *GAPDH* and *HPRT1* gene expression. Values from densitometric analyses of blots were normalized to the total protein content using TGX Stain-Free gel technology (**b** and **c**) or to the abundance of beta-actin (ACTB; **d**). All blots are included in Additional File 3: Fig. S7. The results are presented as a fold change of the respective control values. Data are expressed as the mean ± SEM from 3–4 (**c**) and 6 (**b** and **d**) experiments

Cell-specific gene regulation can be hidden in the response of complete endometrial tissue; therefore, we attempted to uncover the localization and local regulation of SIRT6 in luminal epithelium, the first layer of the endometrium in direct contact with the conceptus and its secretions. As demonstrated in Fig. 2a, immunoreactive SIRT6 protein was localized predominantly in the nucleus, with weaker immunoreaction observed in the cytoplasm of LE cells. Negative control staining showed no specific immunoreaction for SIRT6. Similar to cultured endometrial slices, the treatment of LE cells with CEM or different doses of E2, PGE2, and cytokines did not affect SIRT6 mRNA and protein expression (Fig. 2b, c). Additionally, we investigated whether ovarian steroid hormone P4 is involved in the modulation of SIRT6 expression, but neither P4 alone nor the combined treatment of P4 and E2 affected SIRT6 transcript and protein abundance in LE cells (Fig. 2d).

### RNA sequencing results

RNA-seq was applied to get a picture of SIRT6-dependent transcriptomic changes in the endometrium of early pregnant pigs, and to provide insights into the physiological role of SIRT6 during the implantation period. In total, RNA-seq yielded 136,174,524 and 135,993,985 raw reads in control and UBCS039-treated groups, respectively. After removal of low-quality reads and adapter sequences, 133,372,512 and 125,631,725 clean reads were mapped to the reference porcine genome (Sscrofa11.1), and an average of 92.96% and 92.67% of the reads were mapped to unique genomic regions (Additional file 2: Figure S1). The PCA revealed a high similarity in expression patterns of the biological replicates (Additional file 2: Figure S2). In total, the expression of 17,225 genes was detectable (Additional file 1: Table S3).

### Experiment 3: The effect of SIRT6 activation on differential gene expression in the endometrial tissue

**Fig. 3:**
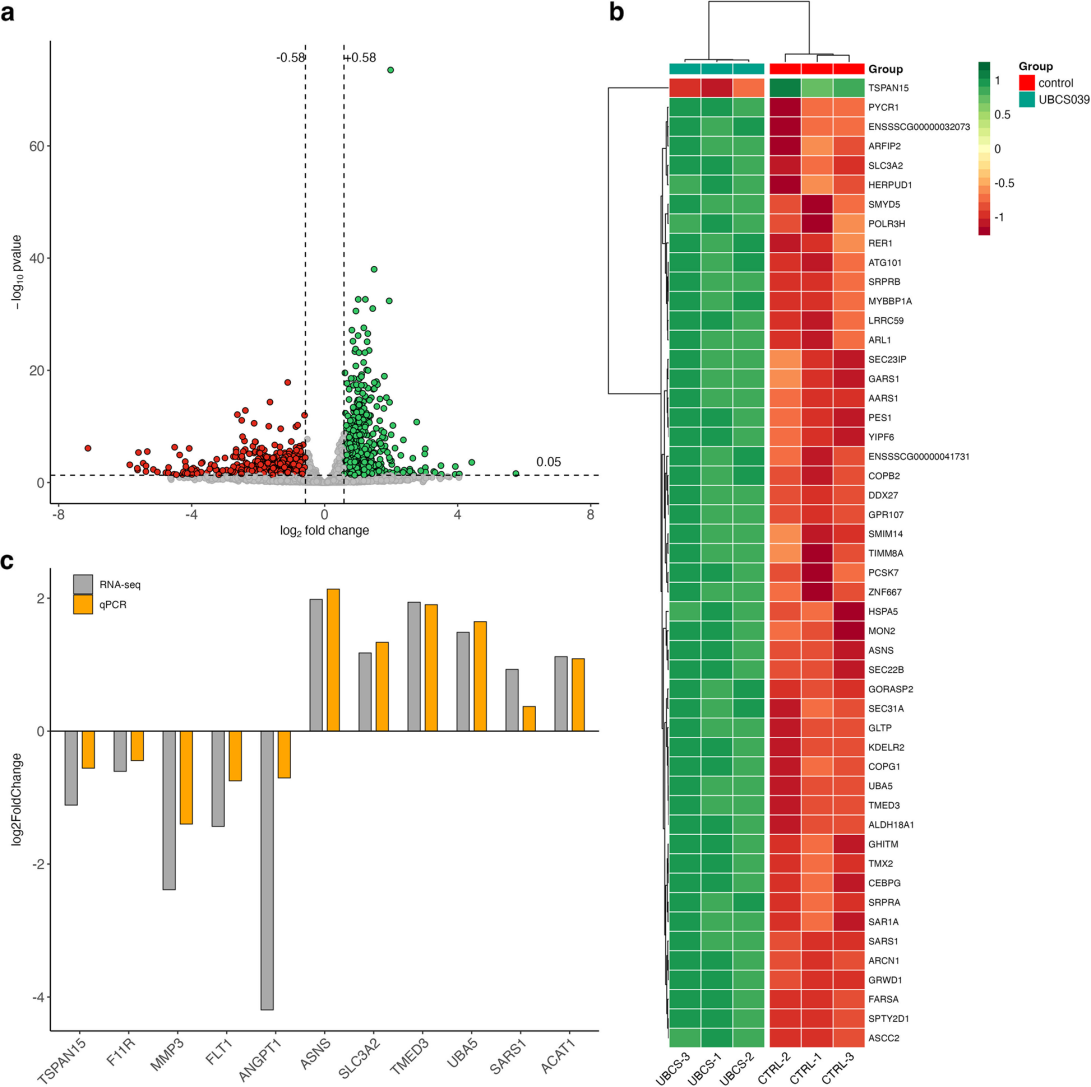
The transcriptome of porcine endometrial tissue in response to SIRT6 activation. **a** Volcano plot with differentially expressed genes (DEGs) in endometrial explants treated with SIRT6 activator, UBCS039, compared to untreated control (adjusted *p*-value < 0.05 and log2 fold change > 0.58). Green dots and red dots represent up-regulated and down-regulated DEGs, respectively. Grey dots represent genes with no significant difference. **b** Heatmap showing hierarchical clustering analysis results according to DEGs after UBCS039 treatment of endometrial explants; the top 50 DEGs are shown. Each row represents one gene, and each column represents a sample. The color scale indicates the relative gene expression level, expressed as a z-score, which represents the number of standard deviations from the mean expression of a given gene. Green indicates higher values in gene expression, and red indicates lower values compared with the respective control (CTRL). **c** The qPCR validation of RNA-sequencing (RNA-seq) data using eleven selected genes. Values from qPCR were normalized to the geometric mean of *GAPDH* and *HPRT1* gene expression. Data are expressed as the log2 fold change of mean expression levels between control and UBCS039-treated endometrial explants (*n* = 3 per group). *TSPAN15*: tetraspanin 15; *F11R*: F11 receptor; *MMP3*: matrix metallopeptidase 3; *FLT1*: fms related receptor tyrosine kinase 1; *ANGPT1*: angiopoietin 1; *ASNS*: asparagine synthetase (glutamine-hydrolyzing); *SLC3A2*: solute carrier family 3 member 2; *TMED3*: transmembrane p24 trafficking protein 3; *UBA5*: ubiquitin like modifier activating enzyme 5; *SARS1*: Seryl-tRNA synthetase 1; *ACAT1*: acetyl-CoA acetyltransferase 1

Analysis of read counts revealed 1544 genes that were significantly changed in endometrial explants treated with UBCS039 for 24 h compared with non-treated control (adjusted *p*-value < 0.05 and log2FC > 0.58) (Additional file 1: Table S4). Among all these DEGs, 788 genes were up-regulated and 756 genes were down-regulated (see a volcano plot; Fig. 3a). Hierarchical clustering of the top 50 DEGs demonstrated clear separation between control and UBCS039-treated samples, confirming consistency of biological replicates (Fig. 3b). Validation of RNA-seq results using qPCR showed strong positive correlation for eleven randomly selected genes (*r* = 0.8877, *p* = 0.0003; Fig. 3c and Additional file 1: Table S5).

### Gene ontology enrichment analysis of DEGs

**Fig. 4:**
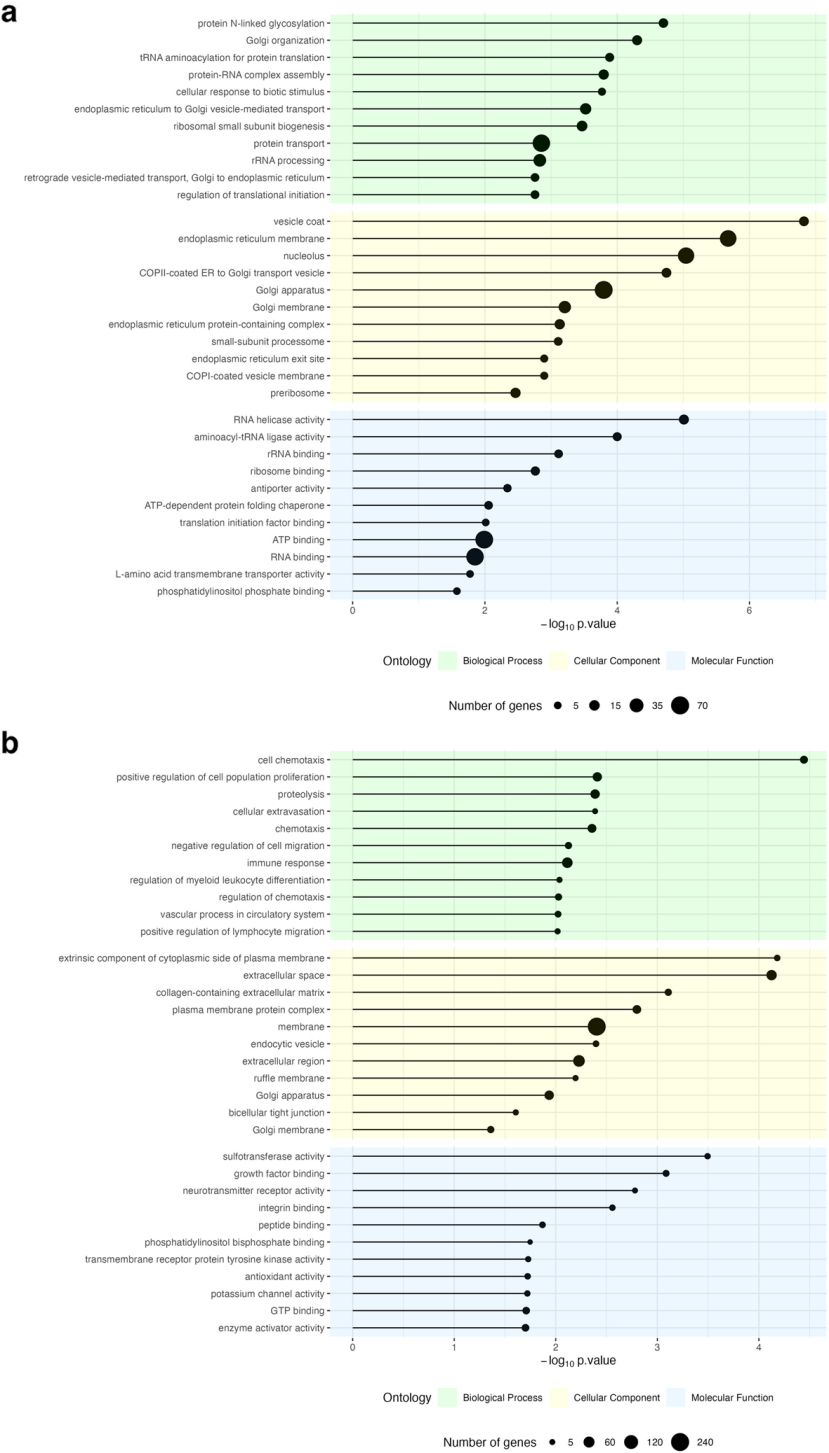
Scatter plots of the top 11 enriched GO terms associated with up- and down-regulated DEGs. The size of the dot reflects the number of genes annotated to the particular GO term (see legend for details)

To identify enriched biological functions among up- and down-regulated DEGs, GO enrichment analysis was performed. The genes were annotated into three GO domains: Biological Process (BP), Cellular Component (CC), and Molecular Function (MF). The overrepresented 11 GO terms from each category are presented in Fig. 4a and b, and all identified functions of DEGs are given in Additional file 1: Tables S6 and S7. Up-regulated genes were assigned to 33 terms of BP, 20 terms of CC, and 14 terms of MF. GO analysis of down-regulated genes revealed 87 significant terms, including 62 BP, 11 CC, and 14 MF. Moreover, a plethora of genes identified in endometrial explants in response to UBCS039 treatment significantly enriched functions attributed, but not limited, to cell proliferation, adhesion, migration, extracellular matrix (ECM) organization, and metabolic processes.

### Protein–protein interaction network analysis for DEGs

Next, all the DEGs were imported into the STRING database to generate a protein–protein interaction (PPI) network, which consisted of 1297 nodes and 2854 edges (Additional file 2: Figure S3). The network showed significant enrichment for PPI (p-value = 1.0e-16), indicating that the proteins are functionally connected. Products of the DEGs were enriched in different pathways, including but not limited to “protein processing in endoplasmic reticulum” (KEGG: 04141), “aminoacyl-tRNA biosynthesis” (KEGG: 00970), “ribosome biogenesis” (KEGG: 03008), “calcium signaling pathway” (KEGG: 04020), and “PI3K-Akt signaling pathway” (KEGG: 04151). MCL clustering analysis of the PPI network identified ten major groups of proteins primarily associated with “rRNA processing” (Cluster 1), “ribosome biogenesis” (Cluster 2), “cellular response to stress” (Cluster 3), “tRNA aminoacylation for protein translation” (Cluster 4), and “Golgi vesicle transport” (Cluster 5). Additionally, 29 genes in Cluster 1 were annotated to “nucleic acid binding”. Interestingly, Cluster 9 was composed of 11 genes significantly associated with “immune response”, “apoptotic signaling pathway”, and “cytokine-mediated signaling pathway”. For details, see Additional file 1: Table S8.

### SIRT6 contributes to PGE2 metabolism in the uterine endometrium

**Fig. 5:**
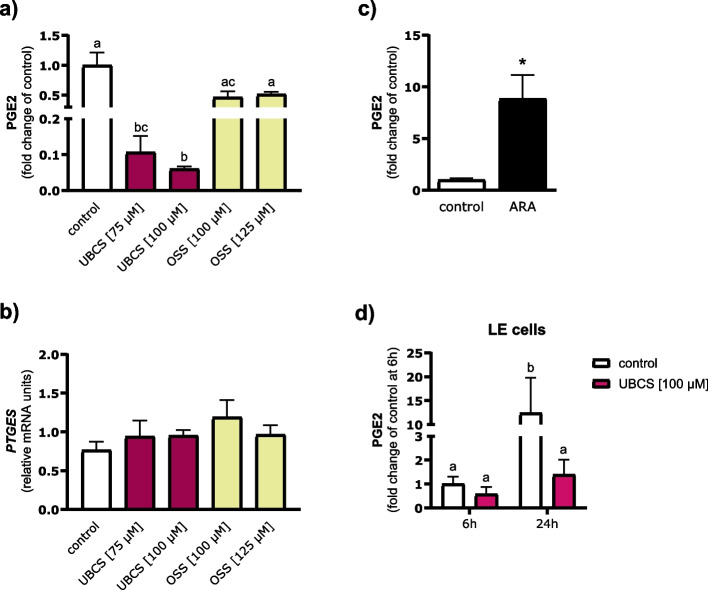
Effect of SIRT6 on prostaglandin (PG) E2 concentration and prostaglandin E synthase (*PTGES*) mRNA expression. **a** and **b** Endometrial slices were exposed to medium only (control) or medium containing SIRT6 activator (UBCS039; 75 and 100 µM) or inhibitor (OSS_128167; 100 µM and 125 µM) for 24 h. **c** Arachidonic acid (ARA; 200 µM) was used as a positive control for PGE2 synthesis. **d** Luminal epithelial (LE) cells were cultured with UBCS039 (100 µM) for 6 and 24 h. PGE2 levels are presented as a fold change of the respective control values. Values from qPCR were normalized to the geometric mean of *GAPDH* and *HPRT1* gene expression. Data are expressed as the mean ± SEM from 4 (**c**, **d**) or 5 (**a**, **b**) experiments. Bars with various letters are significantly different (*p* < 0.05). Asterisk indicates the difference compared with the control (*p* < 0.05)

In the present study, we observed an elevated expression of genes encoding PG-metabolizing enzymes, prostaglandin reductase 1 (*PTGR1*; padj = 0.016) and aldo–keto reductase family 1 member C1 (*AKR1C1*; padj = 0.0013) in the samples treated with UBCS039 (Additional file 1: Table S4). Therefore, we analyzed the concentration of PGE2 in culture media after treatment of endometrial explants with modulators of SIRT6 activity for 24 h. Activation of SIRT6 by UBCS039 efficiently diminished PGE2 level in culture media, reaching 89% and 94% reduction at 75 and 100 μM, respectively (*p* = 0.028 and *p* = 0.002, compared with the control value); while OSS_128167, a selective inhibitor of SIRT6 enzymatic activity, had no effect on PGE2 concentrations (Fig. 5a). A similar suppressive effect was confirmed in LE cells following UBCS039 treatment at a dose of 100 μM, with a tenfold decrease in PGE2 concentration in culture media observed after 24 h of incubation (*p* = 0.046; Fig. 5d). ARA, used as a positive control, showed the expected stimulatory effect on the PGE2 release by endometrial slices (*p* < 0.001; Fig. 5c). To further verify whether SIRT6 is involved in PGE2 metabolism rather than its production, expression of the prostaglandin E synthase (*PTGES*) gene, which encodes the final enzyme in the PGE2 synthesis pathway, was analyzed; neither UBCS039 nor OSS_128167 (Fig. 5b) affected *PTGES* transcript abundance in endometrial strips.

### Effect of SIRT6 on pro‑ and anti‑apoptotic genes in endometrial explants of early pregnant gilts

**Fig. 6:**
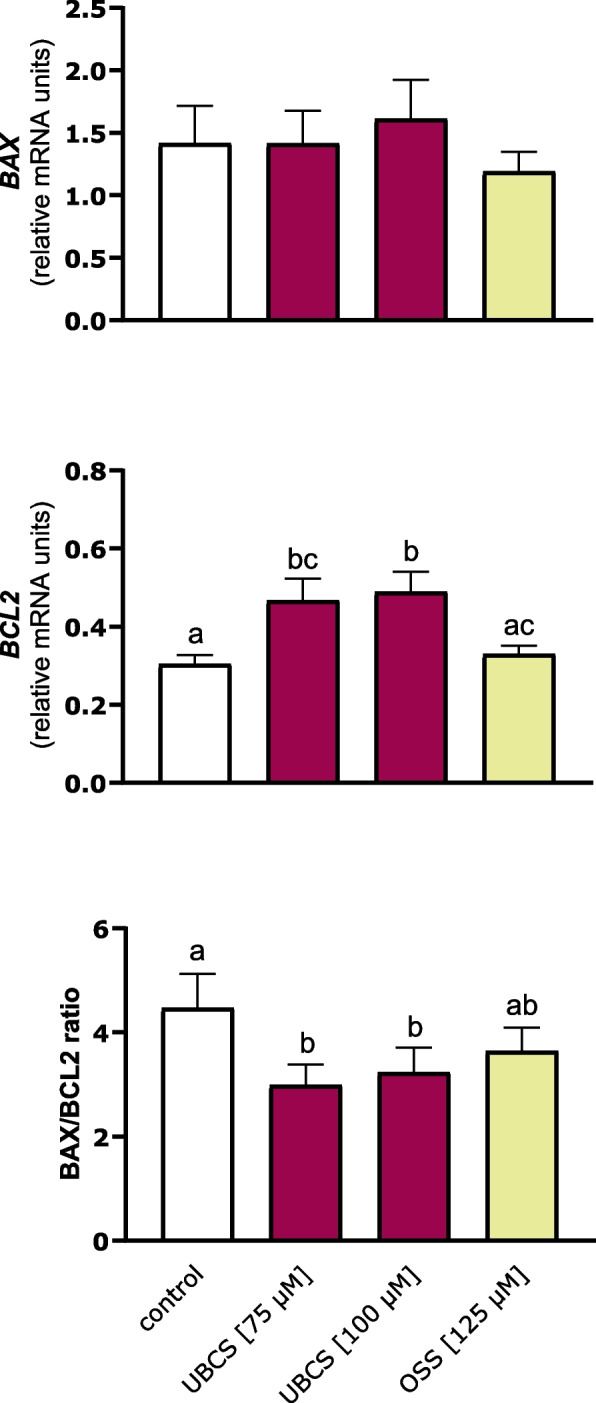
SIRT6 downregulates the *BAX/BCL2* ratio by upregulating the expression of the anti-apoptotic *BCL2* gene. Endometrial slices were exposed to medium only (control) or medium containing SIRT6 activator (UBCS039; 75 and 100 µM) or inhibitor (OSS_128167; 125 µM) for 24 h. Values from qPCR were normalized to the geometric mean of *GAPDH* and *HPRT1* gene expression. Data are expressed as the mean ± SEM from 5 experiments. Bars with various letters are significantly different (*p* < 0.05)

Here, we also found that SIRT6 activation induced the expression of genes related to suppression of apoptosis, e.g., caspase activity and apoptosis inhibitor 1 (*CAAP1*), BCL2 interacting protein 1 (*BNIP1*), and BCL2 like 12 (*BCL2L12*) expression was upregulated in endometrial strips treated with UBCS039 (Additional file 1: Table S4). To extend these findings, we investigated whether modulation of SIRT6 activity affects the expression of other key apoptotic genes in this tissue. Neither activator nor inhibitor of SIRT6 affected the mRNA expression of pro-apoptotic BCL2 associated X, apoptosis regulator (*BAX*) in endometrial explants after 24 h of treatment (*p* > 0.05; Fig. 6), whereas UBCS039 increased the transcript abundance of anti-apoptotic BCL2 apoptosis regulator (*BCL2*) by 60% (*p* < 0.05 and *p* < 0.01 for 75 µM and 100 µM, respectively), as compared with the control. As a consequence of elevated *BCL2* expression, the ratio of *BAX/BCL2* mRNA was downregulated by SIRT6 activator (*p* < 0.05). SIRT6 inhibition by OSS_128167 did not affect *BCL2* mRNA expression or the ratio of *BAX/BCL2* mRNA.

### Effect of SIRT6 on LE cell proliferation and cell cycle progression

**Fig. 7:**
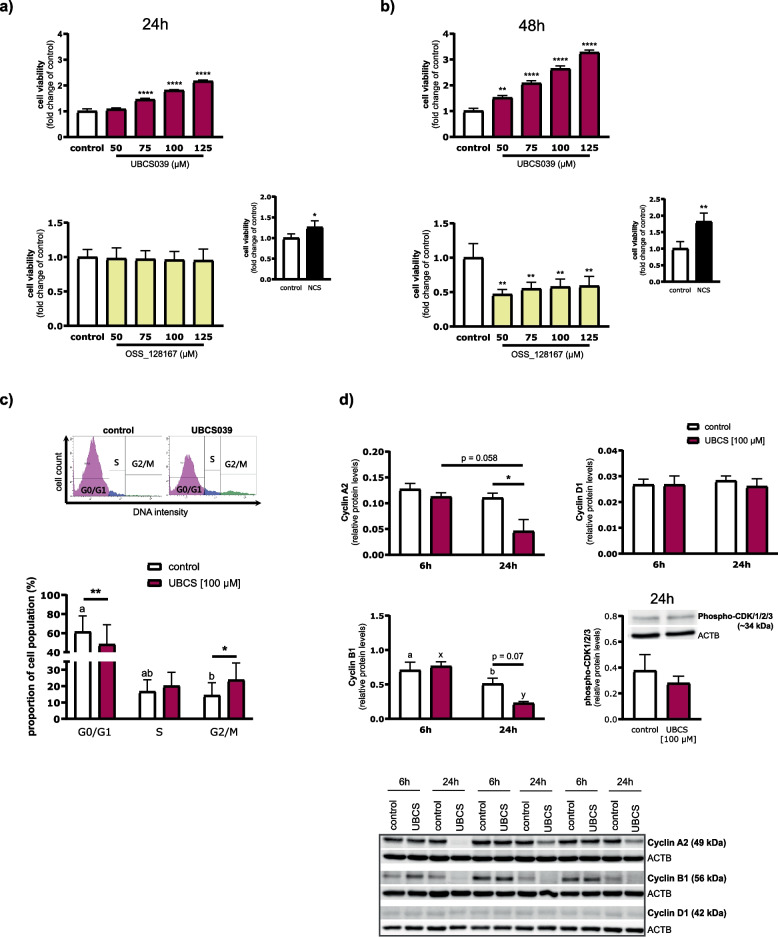
SIRT6 stimulates luminal epithelial (LE) cell proliferation by promoting cell cycle progression. **a** and **b** Effect of SIRT6 on LE cell proliferation. Cells were cultured with SIRT6 activator, UBCS039, or inhibitor, OSS_128167 (50 to 125 µM) for 24 and 48 h. Newborn calf serum (NCS; 20%) was used as a positive control. **c** and **d** Effect of SIRT6 on cell cycle distribution and the expression of essential regulators of cell cycle progression. LE cells were treated with either control media or UBCS039 (100 µM). **c** After 24 h, the percentage of cells in G0/G1, S, and G2/M phases was calculated by fluorescence intensity of incorporated FxCycle Violet Stain in a flow cytometry analysis. Representative images of cell cycle fractions are presented. **d** After 6 and/or 24 h, cell cycle-related protein levels were determined in cell extracts by Western blot. Values from densitometric analyses of blots were normalized to the abundance of beta-actin (ACTB). Western blots used for densitometric quantifications of cyclins are presented. All blots are included in Additional File 3: Fig. S8. Data are expressed as the mean ± SEM (*n* = 3–4). Asterisks indicate differences as compared with the respective control cells (*p < 0.05; **p < 0.01; ****p < 0.0001). Bars with various letters (ab-for control cells; xy-for UBCS039-treated cells) are significantly different among groups

To gain further insights into the physiological relevance of SIRT6, we determined its effect on the viability of LE cells. As shown in Fig. 7a and b, SIRT6 activator dose-dependently enhanced cell viability compared with non-treated cells after 24 (1.5-, 1.8- and 2.2-fold increase for 75, 100 and 125 μM, respectively; *p* < 0.0001) and 48 (1.5- to 3.3-fold increase at 50 to 125 μM; *p* < 0.01) h of incubation. Accordingly, SIRT6 inhibitor, OSS_128167 decreased the number of viable cells at 48 h (~ 50% reduction at 50 to 125 µM; *p* < 0.01) (Fig. 7b). In the presence of NCS, an expected increase in cell proliferation was observed after 24 (*p* < 0.05) and 48 (*p* < 0.01) h of incubation (Fig. 7a and b). To verify the causal relation of SIRT6-enhanced cell viability and cell cycle progression, the cell cycle distribution was analyzed. UBCS039 increased the percentage of LE cells in the G2/M phase (*p* < 0.05), accompanied by decreased cell number at the G0/G1 phase (*p* < 0.01; Fig. 7c) compared to respective control cells. Moreover, among studied cell cycle-regulating proteins, the level of cyclin A2 was markedly diminished (*p* < 0.05) and cyclin B1 expression tended to decrease (*p* = 0.07) after 24 h treatment of LE cells with UBCS039, as compared to respective controls (Fig. 7d). Pronounced reduction of cyclin B1 was evident after 24 h both in control (*p* < 0.05) and UBCS039-treated (*p* < 0.01) cells compared with respective cells incubated for 6 h.

### Effect of SIRT6 on the adhesive properties of LE cells

**Fig. 8:**
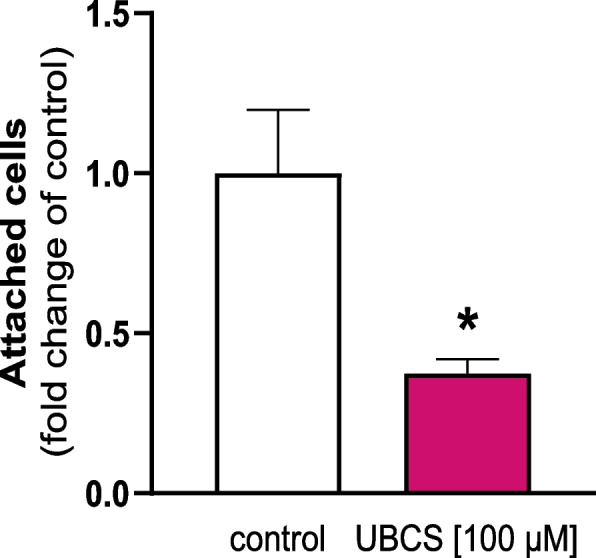
Activation of SIRT6 disrupts the adhesive properties of endometrial luminal epithelial cells. Cells were treated with either control media or UBCS039 (100 µM). Data are expressed as the mean ± SEM (*n* = 3). Asterisk indicates difference as compared with the control cells (*p < 0.05)

Given that SIRT6 downregulates the expression of many adhesion-related genes in endometrial explants (Additional file 1: Table S4), it was also of interest to investigate whether SIRT6 affects the adhesive properties of the uterine epithelium. Indeed, the incubation of LE cells with SIRT6 activator, UBCS039, reduced by 60% the number of cells attached to fibronectin-coated strips compared with nontreated cells (*p* < 0.05; Fig. 8).

## Discussion

This report shows that the SIRT6 protein is present at the utero-conceptus interface during early pregnancy in pigs. It was specifically detected in the uterine epithelium and stroma as well as in the trophoblast tissue. This location suggests a possible role for SIRT6 during the maternal recognition of pregnancy (days 10–12), implantation (days 15–20), and early placentation (day 30). Interestingly, the elevated level of SIRT6 protein in endometrial tissue on day 30 of gestation (compared to day 20) is not due to increased *SIRT6* mRNA expression. According to Kanfi et al*.* [41], reduced proteasomal degradation rather than increased transcription is responsible for stabilizing SIRT6 protein. The ubiquitin proteasome system is also present in the nucleus [42], so it is plausible that nuclear SIRT6 accumulation can result from cytoplasmic stabilization and nuclear leakage, or specific nuclear regulation. Nevertheless, the reduced transcript abundance of SIRT6 after the initial conceptus attachment (after day 15) and transiently lower expression of its protein in endometrial tissue at the end of the formal implantation, when the conceptus firmly adheres to the uterine epithelium, implies that endometrial SIRT6 level can be strictly regulated by the conceptus presence. In pigs, conceptus-derived products, including, but not limited to, E2, PGE2, IL1β, and IFNγ, together with P4 from the corpus luteum, act on the endometrial epithelium to regulate uterine function required for implantation [2, 4]. Thus, in addition to examining the CEM effect, we turned our attention to these molecules to identify factors that may regulate SIRT6 expression in the porcine endometrium. However, none of them affected the expression of SIRT6 mRNA and protein, either in endometrial explants or LE cells. Similarly, P4 and its combination with E2 did not alter SIRT6 levels. Although we failed to confirm conceptus- or P4-dependent modulation of SIRT6 expression, it is possible that other factors of embryonic, ovarian, and endometrial origin are implicated in this regulation; it warrants future investigation.

Further, we identified transcriptomic changes in the porcine endometrium at the time of initial conceptus attachment after SIRT6 activation with UBCS039. The results of this study suggest that SIRT6 supports uterine function by affecting the expression of genes associated with metabolic pathways, mitochondrial activity, and their interactions with other organelles, intracellular protein transport, immune response, endometrial tissue remodeling, as well as the ubiquitination machinery and cell cycle progression, ultimately facilitating cell proliferation.

### SIRT6 modifies the expression of genes related to the metabolism and transport of glucose, amino acids, and polyamines

Concentrations of glucose, a major metabolic fuel for cells, increase in the uterine lumen during early pregnancy in livestock species [43, 44] and are accompanied by increased expression of facilitative glucose transporters of the solute carrier (SLC) 2 A family in the endometrium [43, 45]. In the present study, the activation of SIRT6 elevated *SLC2A10* transcript levels in the endometrial tissue of days 15–16 pregnant gilts. Once delivered, glucose can enter the serine biosynthetic pathway to produce serine and subsequently glycine [44, 46] to meet the metabolic demands of rapidly growing conceptuses. All three genes, *PHGDH*, *PSAT1*, and *PSPH*, which encode key enzymes involved in the de novo serine synthesis pathway, were upregulated by UBCS039. Our findings agree with increased immunoreaction for PHGDH, PSAT1, and PSPH proteins in the porcine luminal epithelium at the time of implantation [46]. Serine can also be taken up via neutral amino acid transporters; here, the mRNA expression of *SLC1A4* and *SLC1A5* was increased in UBCS039-treated endometrium. Thus, our results imply that SIRT6 increases serine availability in the uterus by stimulating its synthesis and uptake. Furthermore, the activation of SIRT6 may drive serine–glycine one-carbon metabolism within the mitochondria by upregulating the expression of serine hydroxymethyltransferase 2 (*SHMT2*) and methylenetetrahydrofolate dehydrogenase 2 (*MTHFD2*) mRNA.

Glutamine is another essential energy source, and its concentration rises in the uterine lumen of gestating pigs [47, 48] and ewes [43], supporting increasing demand for ATP and biosynthetic precursors. It enters cells via transporters belonging to SLC1, SLC6, SLC7, and SLC38 families and is converted to glutamate and then to the TCA intermediate α-ketoglutaric acid by the enzymatic action of glutaminase and aminotransferases, including PSAT1 and glutamic-pyruvic transaminase-2 (GPT2). This pathway generates other amino acids (serine, aspartate), which contribute to the biosynthesis of nucleotides [49]. We showed here that apart from *SLC1A4/5* (mentioned above), also *SLC1A3*, *SLC6A15*, *SLC7A5*, and *SLC38A10* genes were upregulated in endometrial explants treated with UBCS039, possibly to maximize glutamine transport across the uterine epithelium. Together with *PSAT1*, the expression of *GPT2* – known to be expressed in the endometrium of pregnant pigs [5, 46] – was increased in this tissue by SIRT6 activator. Moreover, endometrial genes induced by UBCS039 included: asparagine synthetase (*ASNS*) and asparagine synthetase domain containing 1 (*ASNSD1*). This coincides with increased amounts of asparagine in uterine flushings of pigs between days 10 and 16 of gestation [48]. Notably, glutamine depletion impaired angiogenesis of endothelial cells [50], the defective *ASNS* gene caused G1 arrest [51], and asparagine deprivation induced apoptosis [52]. Thus, our results highlight the crucial role of SIRT6 in glutamine uptake and glutaminolysis for the metabolic reprogramming of the uterine endometrium to support cell bioenergetics during conceptus implantation and early placentation.

Other UBCS039-up-regulated genes of particular interest were ornithine decarboxylase (*ODC1*; based on p-value) and spermidine synthase (*SRM*), which encode rate-limiting enzymes in the polyamine biosynthetic pathway. The activator of SIRT6 also increased the abundance of antizyme inhibitor 1 (*AZIN1*) transcripts but decreased the mRNA expression of ornithine decarboxylase antizyme 2 (*OAZ2*), an ODC1 inhibitor. These suggest that SIRT6 enhances polyamine production. Polyamine transport likely occurs via SLC3A2 [53], whose transcript level was increased by the SIRT6 activator in the present report. In turn, the level of *SLC22A1*, involved in polyamine uptake, was down-regulated in UBCS039-treated samples. We suppose that, similar to what was suggested for SLC22A2 [54], inhibition of the SLC22A1 transporter is necessary when the intracellular level of polyamines is high, to reduce the ability of cells to incorporate these compounds. The mRNA expression of ATP-binding cassette (*ABC*) transporters belonging to the polyamine uptake system was also modulated by SIRT6 in this study. Indeed, SIRT6 might be one of the key players in the process that allows endometrial cells to maintain an appropriate concentration of polyamines during the peri-implantation period. In farm animals, including gestating gilts, spermidine and spermine are the major polyamines synthesized in the endometrium and placenta [53, 55, 56]. In the pig placenta, increased polyamine synthesis between days 20 and 40 of gestation positively correlates with placental weight and fetal growth [56]. Therefore, our findings suggest that SIRT6, by enhancing the polyamine metabolic pathway, may contribute to the development of the endometrial vascular bed to ensure sufficient nutrient supply for fetal growth.

Amino acids, such as glutamine and arginine, as well as polyamines, activate the protein kinase mTOR pathway to support trophoblast cell function in the pig [1, 47]. We previously reported the importance of the activation of the mTOR pathway for PGI2-stimulated endothelial cell proliferation in the porcine endometrium [31]. In this study, the mRNA expression of *mTOR* and eukaryotic translation initiation factor 4E binding protein 3 (*EIF4EBP3*) was stimulated by SIRT6 activator. Our observation coincides with previously reported importance of Sirt6 for the activation of the mTOR pathway; knockdown or inhibition of Sirt6 suppressed PI3K/Akt/mTOR signaling pathway in cancer cells and reduced its oncogenic activity [57]. Furthermore, we identified *RUVBL1* and *RUVBL2* as target genes of SIRT6. These highly conserved AAA + ATPases regulate mTORC1 assembly, lysosomal localization, and activation [58]. All the above, together with increased nutrient metabolism and uptake, suggest that SIRT6 may be essential for the activation of mTOR signaling in the endometrium of pregnant pigs; thus, it can modulate uterine function to ensure conceptus development.

Further, we provide evidence that SIRT6 is a novel activator of endometrial genes encoding aminoacyl-tRNA-synthetases ([aaRS]; e.g., *SARS1*, *GARS1*, *AARS1*, *FARSA*, *HARS1*, *EPRS1*, *VARS1*, *LARS1*, *WARS1*, *NARS1*, *TARS1*, *CARS1*, *MARS1*, *RARS1*, *YARS2,* and *IARS1*), enzymes that catalyze the first step of protein synthesis. Some of these genes, including *WARS* (tryptophanyl tRNA synthetase), are induced in porcine endometrium during early pregnancy [5, 9, 59], and tryptophan metabolism is important for modulating immune tolerance at the time of implantation [59]. Here, we also found that transcript abundance of RNA methyltransferases, such as *METTL1* and *METTL25*, and *ALKBH1*-mediated tRNA demethylation was upregulated by UBCS039. Our results further support the concept that SIRT6 participates in amino acid metabolism, leading to increased protein synthesis in the uterus of early pregnant gilts, and as a result, it may constitute a critical component of various cellular functions, including energy balance and maternal immune tolerance to the embryo presence.

### SIRT6 targets key genes required for mitochondrial activity and energy production

Mitochondria generate ATP through oxidative phosphorylation (OXPHOS) involving five complexes: NADH: ubiquinone oxidoreductase (NDUF, complex I), succinate dehydrogenase (SDH, complex II), ubiquinol–cytochrome c oxidoreductase (UQCC, complex III), cytochrome c oxidase (COX, complex IV), and ATP synthase (complex V) [60]. Our results indicate a role for SIRT6 in the modulation of the OXPHOS system in mitochondria in the uterus of pregnant pigs; UBCS039 increased the expression of *NDUFS1*, *SDHAF1*, and *UQCC4* mRNA, while reducing levels of *NDUFS2*, *NDUFA4L2*, *NDUFA8*, and *COX6C* transcripts. NDUFS1 is known to promote cell viability and migration via OXPHOS in endometrial cells [61]. Similarly, NDUFS2 is essential for the survival, proliferation, and migration of cells by inhibiting mitochondrial cell death [62]. Despite the observed decrease in *NDUFS2* mRNA levels, which may indicate alterations in mitochondrial structure, this change could also play a role in maintaining mitochondrial energetics in the uterus, yet the underlying process remains unclear. Processes of early pregnancy occur in a low-oxygen environment of the uterine lumen [63], and attenuating mitochondrial oxygen consumption involving inhibition of complex I activity appears to be essential to limit oxidative stress. For instance, NDUFA8 is down-regulated in redox imbalance in oocytes [64] and is closely associated with metabolic functions that attenuate oxidative stress [65]. Our results, showing a lower level of *NDUFA8* and hypoxia-induced *NDUFA4L2* genes in UBCS039-treated endometrial explants, together with the observed reduced level of SIRT6 protein in this tissue at the time of formal implantation, could be linked to the need for the temporal limitation of SIRT6 activity, allowing mitochondrial reprogramming to maintain a healthy uterus under the hypoxic conditions. On the other hand, SIRT6 induced the translation activator *TACO1* gene; thereby, it may stimulate the synthesis of mitochondria-encoded COX1 and play a role in energy production. Moreover, transcript abundance of nuclear-encoded *COX6C*, which triggers the mitochondrial apoptotic pathway [66], was diminished by UBCS039. Overall, the activation of SIRT6 led to increased expression of many genes known for their association with mitochondrial quality control and function. SIRT6, by inducing genes for mitochondrial methyltransferases, *MRM2* and *MRM3*, may modulate mitochondrial protein function in the uterus. SIRT6 also stimulated the expression of genes encoding mitochondrial *ALDH1L2* folate regulatory enzyme as well as *ALDH18A1* and *PYCR1*-mediated proline synthesis, whereas it reduced the transcript levels of *ALDH4A1* involved in proline degradation. We can assume that SIRT6 may exert metabolic roles in the uterus of pregnant pigs by modulating the levels of aldehyde dehydrogenase family members located in the mitochondria. Additionally, SIRT6 induced the expression of the *ACAD11* gene, which encodes a member of the acyl-CoA dehydrogenase family involved in β-oxidation of fatty acids and is also required for efficient OXPHOS and cell survival under metabolic stress [67]. Among other genes associated with lipid metabolism, nuclear *ACSS2* was diminished in response to UBCS039. Of interest, *ACSS2* knockdown in human endometrial cells promoted cell proliferation [68]. All these data point to SIRT6 as a central component of the transcriptional regulatory machinery that coordinately controls the energy-generating functions of mitochondria in accordance with the uterine metabolic demands imposed by changing physiological conditions during pregnancy.

### SIRT6-dependent expression of hub genes involved in mitochondria-organelle crosstalk

In the current study, we show that many DEGs are highly enriched in GO terms associated with intracellular protein transport and trafficking. In particular, SIRT6 activation up-regulates a group of genes encoding proteins involved in vesicular transport, such as coat protein complex (COP) I and COPII components and cargo receptors (*SAR1A*, *SAR1B*, *SEC23A*, *SEC23B*, *SEC24A*, *SEC24D*, *SEC13*, *SEC31A*, *COPB1*, *COPB2*, *COPE*, *COPZ2*, *LMAN1*, and *MIA3*). SIRT6 also elevates the endometrial abundance of *STX5*, *SEC22B*, *GOSR2*, and *YKT6* transcripts that control both anterograde and retrograde Golgi trafficking. These transports must be precisely coordinated to maintain membrane homeostasis and organelle composition [69]. SIRT6 seems to control these mechanisms in the porcine uterus. The mRNA expression of *SEC23IP,* known to be involved in cholesterol trafficking from the plasma membrane to mitochondria for steroidogenesis [70], was also increased in endometrial tissue incubated with UBCS039. Similarly, the mitochondrial ATPase *ATAD3A* gene was upregulated by UBCS039. Of note, ATAD3A is essential for cholesterol transport from intracellular stores into mitochondria [71] and promotes the proliferation of epithelial cells via the mTOR pathway [72]. Thus, our results provide evidence linking SIRT6 to the maintenance of mitochondrial structure, cholesterol homeostasis, and cell survival. Furthermore, SIRT6, by inducing the expression of genes encoding translocases of inner and outer mitochondrial membranes (*TIMM* and *TOMM40*), members of the Hsp70 (*HSPA*) and Hsp90 (*HSP90B1*) families of molecular chaperones, and HSPA binding protein 1 (*HSPBP1*), may be essential for the import of protein precursors into mitochondria of endometrial cells. In parallel, ER stress-related gene *SERP1* and a few transcripts encoding components of the signal recognition particle (SRP) complex, such as *SRPRA*, *SRPRB*, and *SRP54*, responsible for the translocation of protein across the ER membrane, displayed higher abundance in endometrial tissue treated with UBCS039. The expression of genes belonging to the Rab family of small GTPases, such as *RAB43*, *RAB39B*, and *RAB27B*, was also elevated in these samples. Recent findings [73] suggested that the RAB27 protein, colocalized in luminal epithelium and trophoblast of day 16 pregnant gilts, may govern extracellular vesicle-mediated transport at the embryo–maternal attachment sites. Thus, it can be assumed that SIRT6 establishes and mediates cell-to-cell communication between the endometrium and developing conceptuses in this species.

### DEGs involved in endometrial tissue remodeling

Extensive remodeling of the endometrial matrices is necessary to facilitate conceptus attachment and to increase utero-placental associations [74, 75]. Intriguingly, results of the present work revealed decreases in the expression of many genes related to ECM architecture, cell adhesion, and cell–cell junctions in endometrial tissue of gestating gilts after activation of SIRT6. These genes encode, among others: (a) the major components of basement membrane, such as collagen IV (*COL4A1*, *COL4A2*) and XV (*COL15A1*), laminin (*LAMC1*, *LAMB1*), and nidogen (*NID2*); (b) proteins regulating ECM and cytoskeleton (e.g. *COL3A1*, *LOX*, *LOXL1*, *CDH3*, *CDH5*, *PCDH12*, *CLDN5*); (c) the adhesion molecules, including integrins (*ITGA8*, *ITGA9*, *ITGB3*, *ITGB5*); (d) matrix metallopeptidases (*MMP1*, *MMP3*, *MMP15*, *MMP28*), a disintegrin and metalloproteinase (*ADAM19*), and ADAM with thrombospondin motifs (*ADAMTS1*, *ADAMTS2*, *ADAMTS4-7*, *ADAMTS9*, *ADAMTS12*); and (e) protein components of tight junctions, such as *CLDN5*, *GJA5*, junctophilin 3, and *F11R*.

Collagen IV and laminin immunoreactivities of bovine endometrial epithelium remarkably declined at the time of implantation but reappeared by day 30 of gestation in early placenta development [76]. In pigs, *COL4A*, *LAMC*, and *NID2* genes were induced in the endometrium during maternal recognition of pregnancy and conceptus adhesion (days 12–16) compared with the corresponding periods of the estrous cycle [5, 6, 9]. There is limited data, however, on whether the level of these matrix molecules in the porcine uterus changes after implantation throughout the pregnancy. Chen et al*.* [7] found that in the attachment sites of the porcine endometrium, the expression of *COL15A1* mRNA was reduced at the time of formal implantation compared to early placenta formation, suggesting time-dependent production and elimination of collagen as implantation proceeds. It is consistent with findings from other species [76, 77]. In light of these data, we suggest that SIRT6 may participate in the modification of the composition of the basal lamina necessary for maintaining the integrity of the uterine epithelium throughout pregnancy.

The induction of *ITGB3* and *ITGB5* genes was found in porcine uterine epithelium during initiation of embryo attachment [9], and integrin heterodimers ITGAV/ITGB3 and ITGAV/ITGB5 were shown to stabilize trophoblast cell adhesion to luminal epithelium in humans and pigs [78–80]. In addition, immunostaining of the attachment sites in the porcine uterus identified intense aggregates of ITGB5 (beginning day 20) and ITGB3 (beginning day 24) that were absent at the apical surface of luminal epithelium at day 24 non-attachment sites [80]. A similar observation was made for *CDH3* (p-cadherin) and *CDH5* mRNA, whose expression levels were upregulated in endometrial epithelial cells treated with extracellular vesicles derived from uterine flushings of early pregnant pigs [81]. We assume that the inhibitory effect of SIRT6 on these adhesion-related genes (current study) may result from our experimental model in which endometrial explants, detached from the trophoblast tissue, were exposed to SIRT6 activator in the absence of embryonic signals. Nevertheless, SIRT6 is associated with impaired cell adhesion ability [82]. In fact, we observed that the SIRT6 activator diminished the adhesive properties of LE cells, which is consistent with the overrepresentation of functional categories related to the regulation of cell adhesion for genes with lower expression in UBCS039-treated endometrial tissue. Thus, it is possible that the temporal downregulation of SIRT6 protein on day 20 of gestation (present study) is required for the firm attachment of trophoblast. On the other hand, the abundance of *ITGA9* transcripts (reduced by UBCS039 in our study) was dramatically decreased in endometrial cells between days 12 and 14 of pregnancy in the pig [9], implying a possible role of SIRT6 in a complex regulation of integrin genes in the uterus at the beginning of conceptus adhesion. Of note, the number of p-cadherin-positive stromal cells in the bovine uterus was reduced as the pregnancy progressed [83]; so far, there is no study on this aspect in the pig.

Our study is the first to demonstrate that SIRT6 is involved in restricting the expression of several MMPs in the uterus. This agrees with previous findings that MMP1 and MMP3 levels are specifically regulated by SIRT6; that is, silencing of the *SIRT6* gene in mesenchymal cells increased *MMP1* mRNA expression and prompted MMP1 and MMP3 secretion, inducing ECM degradation [84–86]. Accordingly, endometrial expression of *ADAM* and *ADAMTS*, as well as cathepsins (*CTS*) and their inhibitor cystatin C (*CST3*), all of which are responsible for the activation of MMPs, was reduced by the SIRT6 activator. A similar suppressive effect of UBCS039 was observed on the *CTSL1* gene; its protein product is particularly effective in degrading ECM proteins in species with invasive implantation [87]. Given that the action of MMPs and proteases is potentially destructive, their expression and activity at the maternal-placenta interface must be tightly controlled to maintain the functional epithelium and regulate the paracellular transport in the pig, a species with a true epitheliochorial placenta. In view of this, SIRT6 is probably essential to prevent any excessive endometrial ECM degradation at the time of embryo attachment.

Previous studies described distinct changes in the expression and distribution of several junction-associated molecules in the endometrium of pigs [5, 7, 9, 74], and some of these changes are under hormonal control [74]. Our results propose a novel SIRT6-dependent regulatory mechanism that may explain reduced transcript abundance of *GJA5*, junctophilin 3, and *F11R* noted in the endometrium of early pregnant gilts [5]. However, owing to the importance of epithelial polarity and junctional complexes in the establishment of gestation, it is difficult to explain the biological significance of this action. Nevertheless, in accordance with our findings, another omic analysis of porcine endometrium has also shown overrepresentation of both up- and down-regulated genes related to cell adhesion and tight junction at the time of embryo implantation [8].

### SIRT6 influences the expression of genes related to inflammatory reactions in the endometrium

Many genes related to immune response, including those encoding cytokines and chemokines, are overrepresented in the porcine endometrium during the peri-implantation period [7, 9, 88]. On the other hand, excessive expression of pro-inflammatory cytokines, IFNγ and TNF in the endometrium is associated with arrested conceptus development [89]. Thus, tightly controlled expression of immune-related genes is critical for pregnancy progression. Our data highlights the importance of SIRT6 in the inflammatory reaction of the endometrium. For instance, the abundance of IFN-stimulated gene (*ISG20L2*) and *IFRD2* transcripts was enhanced, while the expression of *JAK3*, *STAT5A*, and *IRF2BPL* genes was diminished by SIRT6, suggesting its role in the IFN response pathway and immune responses supporting endometrial remodeling for successful implantation [90]. Concomitantly, activation of SIRT6 elevated mRNA expression of IL-6 family cytokine oncostatin M (*OSM*), reported to support embryo implantation [91] and to inhibit inflammatory stress in response to IFNγ- and GM-CSF-induced p-STAT [92]. Along with the SIRT6-induced *OSM* gene, the abundance of *CSF2*, *CSF3*, and *CSF2RB* transcripts was reduced. Similarly, the activation of SIRT6 modulated the mRNA expression of *TNF*, TNF superfamily members (*TNFSF9*, *TNFSF12*), and their receptors (*TNFRSF11B* and *TRADD*). Moreover, the present work revealed decreases in the number of endometrial expression of both pro- and anti-inflammatory interleukins, chemokines, and their receptors under the influence of UBCS039. Such results are consistent with the notion that SIRT6 may have distinct, even opposite, actions in regulating inflammation as shown in vascular endothelial [17] and cancer [93] cells. It indicates that endometrial SIRT6 acting as a maternal immune-tolerant factor may balance pro- and anti-inflammatory responses for maintaining a healthy uterus and promoting survival of the early pig conceptuses.

### SIRT6-dependent factors controlling ubiquitin–proteasome system, cell cycle, cell proliferation, and apoptosis in the endometrium

The ubiquitination machinery plays a critical role in DNA replication, apoptosis, and cell cycle progression via precise spatio-temporal control of proteins, including cyclins, CDKs, and cyclin-dependent inhibitors (CKIs) [94]. In the present study, activation of SIRT6 raised the uterine expression of genes encoding ubiquitin-specific proteases (*USP1*, *USP5*, *USP36*, *USPL1*) that can be involved in cell proliferation by promoting cyclin D1 deubiquitination and stabilization [95]. We also demonstrated that SIRT6 may regulate the cell cycle in the endometrium by modulating the expression of F-box genes (*FBXW7*, *FBXOs*); some of them can directly regulate cyclins or CKIs [94]. Additionally, we noted changes in the expression of many other genes directly involved in proper cell cycle progression: *CHEK1*, *CDK2AP2*, *CDCA3*, *CCPG1*, *HIRA*, *CDC40*, *MAD1L1*, *GAK*, and *CENPM* were upregulated, while the cyclin-dependent kinase inhibitor *CDKN2B* was reduced in endometrial explants following SIRT6 activation. Moreover, activation of SIRT6 led to an increase in mRNA expression of ubiquitin fold modifier 1 (*UFM1*) and UFM1-activating enzyme (*UBA5*) that mediate UFMylation, a ubiquitin-like modification, essential for maintaining cell cycle homeostasis [96]. Several other genes for enzymes belonging to the ubiquitin–proteasome system, such as ubiquitin-conjugating enzyme *UBE2QL1* and ubiquitin-ligases, showed altered expression in UBCS039-treated endometrial tissue. Of relevance, the ANAPC16, whose mRNA expression was diminished by SIRT6, is a component of the APC/C complex that is active mainly in the G1 phase of the cell cycle and functions as the key antagonist of mitotic CDKs [94].

Our further observation showed a reduced number of LE cells in the G0/G1 stages after UBCS039 treatment, whereas the expression of cyclin D1, which regulates G1 progression, was unaffected. Phospho-CDK1/2/3 protein levels in these cells remained unchanged, suggesting CDK activity was maintained. SIRT6 may, however, regulate the cell cycle by inducing degradation of cyclin A2 and B1—a step necessary for mitotic exit [97], thereby accelerating progression through the G2/M phase and cell proliferation. In support, the activation of SIRT6 enhanced the proliferating activity of LE cells, whereas OSS_128167 (a SIRT6 inhibitor) markedly diminished the number of viable cells. Our findings agree with Ardestani and Liang [13], who demonstrated that SIRT6 may play a role in the mitosis of HeLa cells. Furthermore, we observed that the endometrial transcript abundance of *PCNA*—a marker of cell proliferation—was increased by UBCS039, providing additional evidence of the pro-survival effect of SIRT6 in the uterine tissue. In line with this, SIRT6 activation reduced the mRNA expression of *ssc-miR-143*, previously shown to inhibit endometrial stromal cell proliferation [98].

Cell proliferation and apoptosis are tightly regulated in the uterus, as their balance is crucial for successful implantation and normal placentation. We identified genes positively associated with apoptosis (e.g., *ECSCR*, *RASSF2*, *RAB*, *RHOJ*, *CIDEA*, *DAPK2*) whose expression was downregulated by UBCS039. Concurrently, the expression of genes encoding *BNIP1* and BCL2 like 12, which interact with the BCL-2 family of anti-apoptotic proteins, was induced. Further experiments expanded these findings, showing that activation of SIRT6 in endometrial tissue led to a dose-dependent increase in *BCL2* mRNA expression that resulted in a decreased ratio of *BAX/BCL2*. The role of SIRT6 in apoptosis is tissue- or cell-specific and context-dependent, and SIRT6 can act as both a positive and a negative regulator of this process. For instance, it promotes the apoptosis of human endometrial cancer cells [25], while reduces the number of apoptotic ovarian cells in mice [19]. Our results help to clarify the limited understanding of the regulation of apoptosis in the porcine uterus during gestation, highlighting SIRT6 as a suppressor of this process.

### SIRT6 may contribute to PGE2 and steroid metabolism

Actions of PGs at the maternal-conceptus interface are essential and tightly regulated for the establishment and maintenance of pregnancy [1]. Lappas [17] proposed that SIRT6 may affect PG production; knockdown of *SIRT6* in HUVEC cells increased PG-endoperoxide synthase (*PTGS2*) mRNA expression and subsequent PGE2 and PGF2α release. Our findings showed that SIRT6 activation in endometrial strips and LE cells reduced the levels of PGE2 in culture media. However, we found that this action may result from increased PGE2 metabolism rather than from decreased synthesis; while endometrial expression of *PTGS2* and PGE2 synthase (*PTGES*) mRNA was unaffected by modulation of SIRT6 activity (either by UBCS039 or OSS_128167), the abundance of genes for PGE2 metabolism (*PTGR1* and *AKR1C1*) was elevated in response to SIRT6 activator. In pigs, enzymes involved in PGE2 metabolism are expressed in the endometrium in a stage of pregnancy-specific manner [99]. Our study provides new insights into the contribution of SIRT6 to PGE2 metabolism in the uterus of pregnant pigs. Furthermore, SIRT6, by inhibiting the expression of the *CYP11A1* gene, may be crucial for the control of steroid synthesis. Excessive *CYP11A1* mRNA expression may impair the placentation process, contributing to pregnancy complications [100]. In addition, SIRT6 appears to be essential for P4 metabolism and signaling in the uterus, because the expression of the *SRD5A1* gene, which encodes steroid 5α-reductase 1, was reduced in endometrial explants treated with UBCS039. Increased SRD5A1 activity in the endometrium may limit the actions of P4 by reducing the availability of its active form [101].

The major limitation of this study is the inability to identify genomic regions enriched with SIRT6 and to detect its physical association with specific histone tails in the porcine endometrium during implantation. However, none of the available SIRT6 antibodies are suitable for chromatin immunoprecipitation. Therefore, further research using alternative advanced techniques (e.g., ATAC-seq, proteomics) that could provide insights into these interactions is warranted.

## Conclusions

**Fig. 9:**
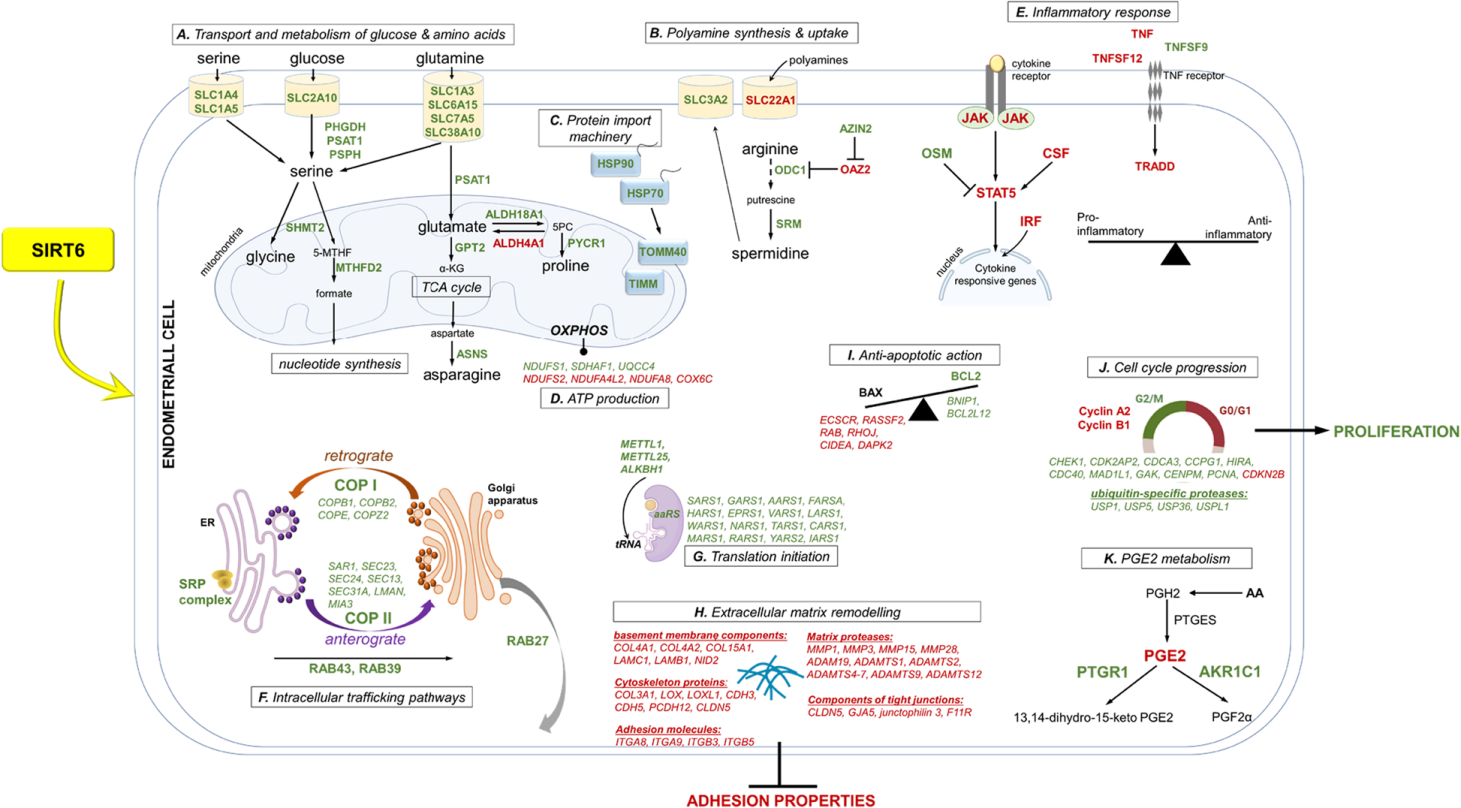
Graphical summary of the molecular and biological effects of SIRT6 in the porcine peri-implantation endometrium. Molecules and cellular processes affected by SIRT6 are indicated in green (stimulation) and red (inhibition). **A** SIRT6 facilitates the transport of serine, glucose, and glutamine via the SLC transporters for metabolism; an intermediate of glycolysis is converted into serine by enzymatic actions of PHGDH, PSAT1, and PSPH, and then into glycine and formate via SHMT2 and MTHFD2; glutamine is converted to α-ketoglutarate (α-KG) by PSAT1 and GPT2, for entry into the TCA cycle. This releases aspartate, which is converted to asparagine via ASNS. SIRT6 may drive the biosynthesis of proline from glutamate by enhancing the activity of ALDH18A1 and PYCR1, and by inhibiting ALDH4A1. **B** SIRT6 induces the polyamine-spermidine pathway by up-regulating ODC1 and SRM expression and affecting polyamine uptake and transport. **C** SIRT6 facilitates the import of protein precursors into mitochondria. **D** SIRT6-affected genes are related to the oxidative phosphorylation (OXPHOS) system and ATP production. **E** SIRT6 may balance pro- and anti-inflammatory responses; e.g., it inhibits the cytokine-dependent JAK/STAT5/IRF pathway and modulates the levels of anti-inflammatory OSM and pro-inflammatory CSF. SIRT6 regulates the levels of TNF family members and inhibits TRADD, a TNF receptor 1-associated signal transducer. **F** SIRT6 is essential for intracellular trafficking by facilitating (i) the translocation of proteins across the ER membrane involving the SRP complex, (ii) anterograde and retrograde Golgi trafficking involving COPII and COPI components, and (iii) extracellular vesicle-mediated transport involving RAB proteins. **G** SIRT6 controls the initiation of translation by inducing the expression of aminoacyl-tRNA-synthetases (aaRS), *METTL*, and *ALKBH1*. **H** SIRT6 affects extracellular matrix remodeling and impairs adhesion of luminal epithelial cells.** I** Anti-apoptotic action of SIRT6. **J** SIRT6 accelerates cell cycle progression through the G2/M phase and promotes cell proliferation. **K** SIRT6 induces prostaglandin (PG) E2 metabolism. The full names of depicted molecules are listed in Additional file 1: Table S3

The present study shows that SIRT6 is present in the endometrium, with a differential expression pattern throughout early pregnancy, and in the trophoblast tissue of the pigs. This report also suggests a potential role for SIRT6 in regulating uterine biological functions around the time of implantation in this species; however, the study is limited by the lack of mechanistic in vivo models to definitely confirm this conclusion. Nevertheless, SIRT6 regulates the expression of endometrial genes related to metabolism (of nutrients, steroids, and PGE2), intracellular protein transport and trafficking, mitochondrial function, ECM organization, inflammatory pathways, the ubiquitin–proteasome system, the cell cycle, apoptosis, and other regulatory cues (Fig. 9). Moreover, SIRT6 differently modulates the adhesive properties and proliferating activity of LE cells. Our research offers novel insights into the regulation mechanism of peri-implantation events in pigs, and places SIRT6 in a central role of reproductive functions critical to the establishment and maintenance of pregnancy. Thus, SIRT6 could be considered as a new, attractive target for developing the future management strategy to improve the survival of the early conceptuses.

## Supplementary Information

Additional file 1: Table S1. Full names of genes and the ID numbers of TaqMan assays used for qPCR. Table S2. Details of primary antibodies used in Western blot. Table S3. A full list of genes expressed in porcine endometrial explants in response to UBCS039 treatment. Table S4. A list of DEGs in endometrial explants in response to UBCS039 treatment compared to control. Table S5. Real-time PCR validation of RNA-seq data. Table S6. Enriched Gene Ontology (GO) terms of up-regulated DEGs. Table S7. Enriched Gene Ontology (GO) terms of down-regulated DEGs. Table S8. The top ten clusters of protein-protein interaction sub-network for all DEGs generated using the STRING database.

Additional file 2: Figure S1. Alignment rates within samples. Figure S2. Graphical plot of principal component analysis (PCA) for data quality control. Figure S3. Predicted protein-protein interaction (PPI) analysis.

Additional file 3: Figures S4-S8. Original images for Western blots.

## Data Availability

The raw sequencing reads were deposited as FASTQ format in the European Nucleotide Archive (ENA) database and can be accessed via accession number PRJEB101134. Other datasets analyzed during the current study are available in the Repository for Open Data (RepOD), https://doi.org/10.18150/2UKG1E.

## Abbreviations

α-KG: α-Ketoglutarate
ARA: Arachidonic acid
BAX: BCL2 associated X, apoptosis regulator
BCL2: BCL2 apoptosis regulator
BP: Biological Process
BSA: Bovine serum albumin
CC: Cellular Component
CDK: Cyclin-dependent kinase
CEM: Conceptus-exposed medium
CKI: Cyclin-dependent inhibitor
COP: Coat protein complex
DEGs: Differentially expressed genes
E2: Estradiol 17β
ECM: Extracellular matrix
EIA: Enzyme immunoassay
GO: Gene ontology
IFNγ: Interferon γ
IL1β: Interleukin 1β
LE cells: Luminal epithelial cells
MF: Molecular Function
NCS: Newborn calf serum
OSS_128167: Sirtuin 6 inhibitor
OXPHOS: Oxidative phosphorylation
P4: Progesterone
PCA: Principal component analysis
PFA: Paraformaldehyde
PGE2: Prostaglandin E2
PPI: Protein-protein interaction
PTGES: Prostaglandin E synthase
RNA-seq: RNA-sequencing
SIRT6: Sirtuin 6
STRING: Search tool for the retrieval interacting genes
UBCS039: Sirtuin 6 activator
