# Supplementary material for: Expression, regulation, and multifaceted molecular and biological functions of sirtuin 6 in the porcine endometrium during early pregnancy
Source: Cell Commun Signal. 2026 Feb 4;24:158. doi: 10.1186/s12964-026-02699-1 (PMC12958535; doi:10.1186/s12964-026-02699-1)
Supplement: Supplementary file 2 — Additional file 2: Figure S1. Alignment rates within samples. Figure S2. Graphical plot of principal component analysis (PCA) for data quality control. Figure S3. Predicted protein-protein interaction (PPI) analysis. [file 12964_2026_2699_MOESM2_ESM.docx]

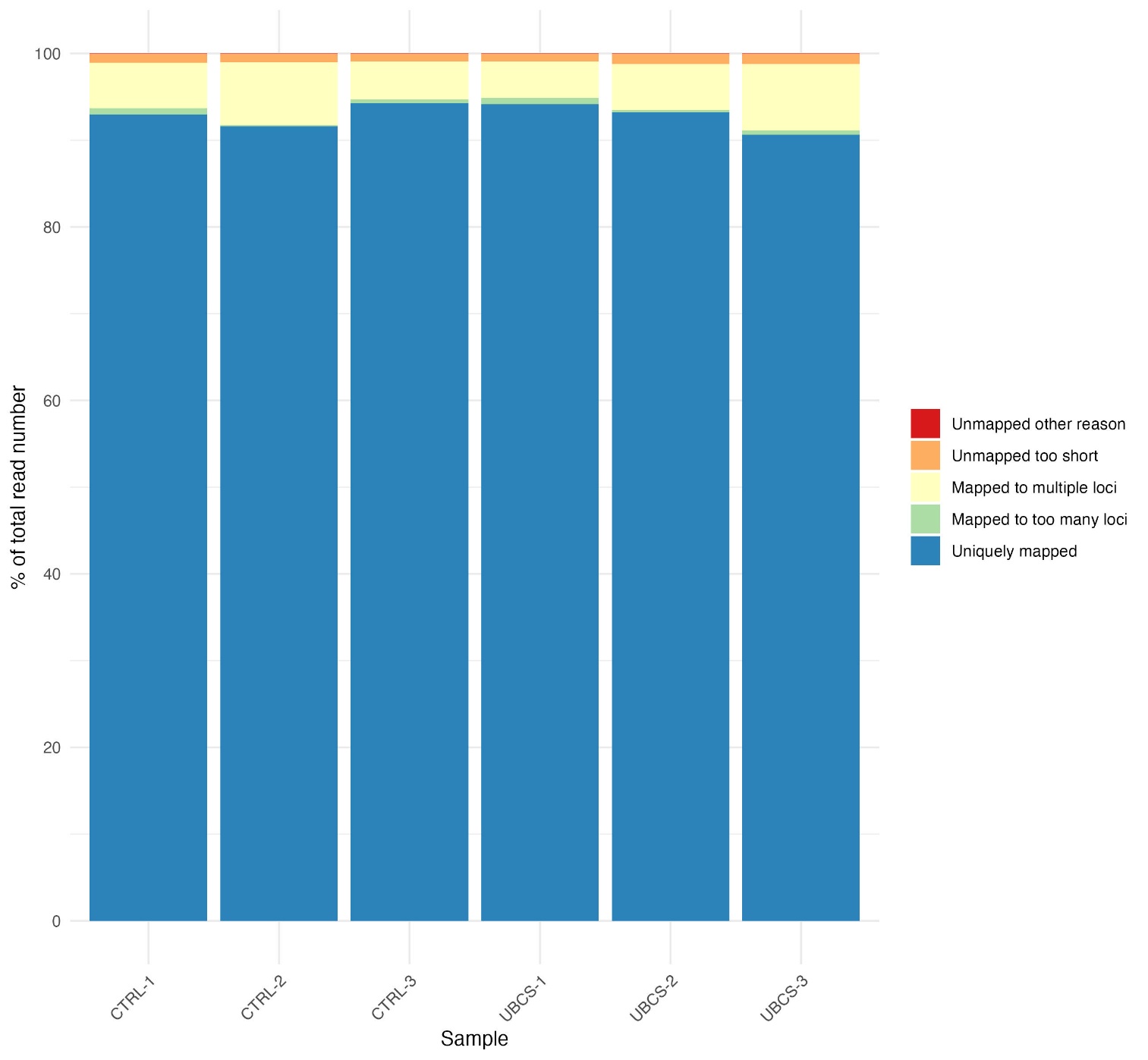


**Figure S1.** Alignment rates within samples. Each bar depicts a fraction of sample's reads that were uniquely mapped, mapped to too many loci, mapped to multiple loci, were unmapped due to short length or other reasons.


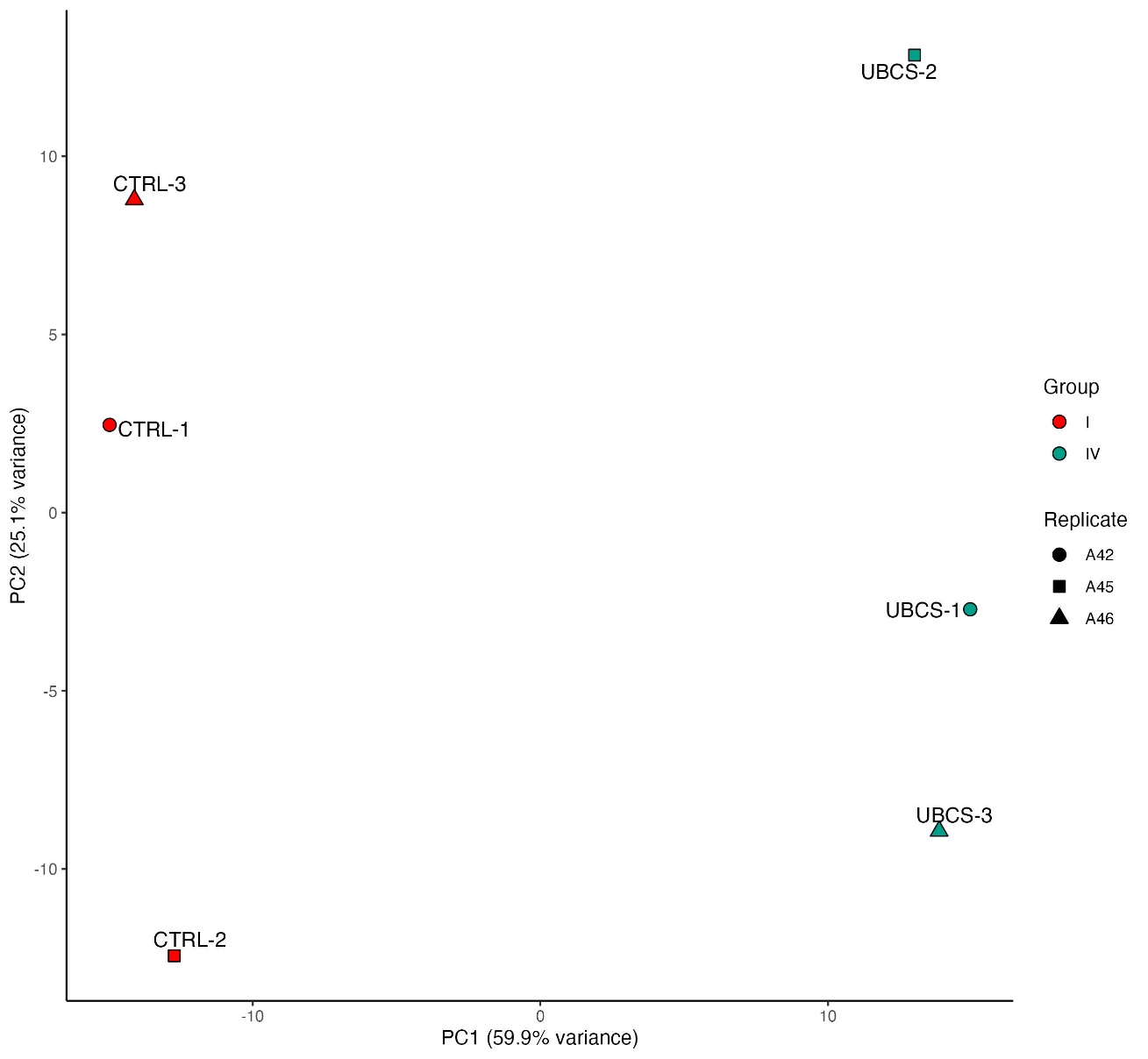


**Figure S2.** Graphical plot of the first and second principal components (PC1 and PC2) affecting the gene expression pattern in three biological replicates of control and UBCS039-treated endometrial explants of early pregnant gilts.


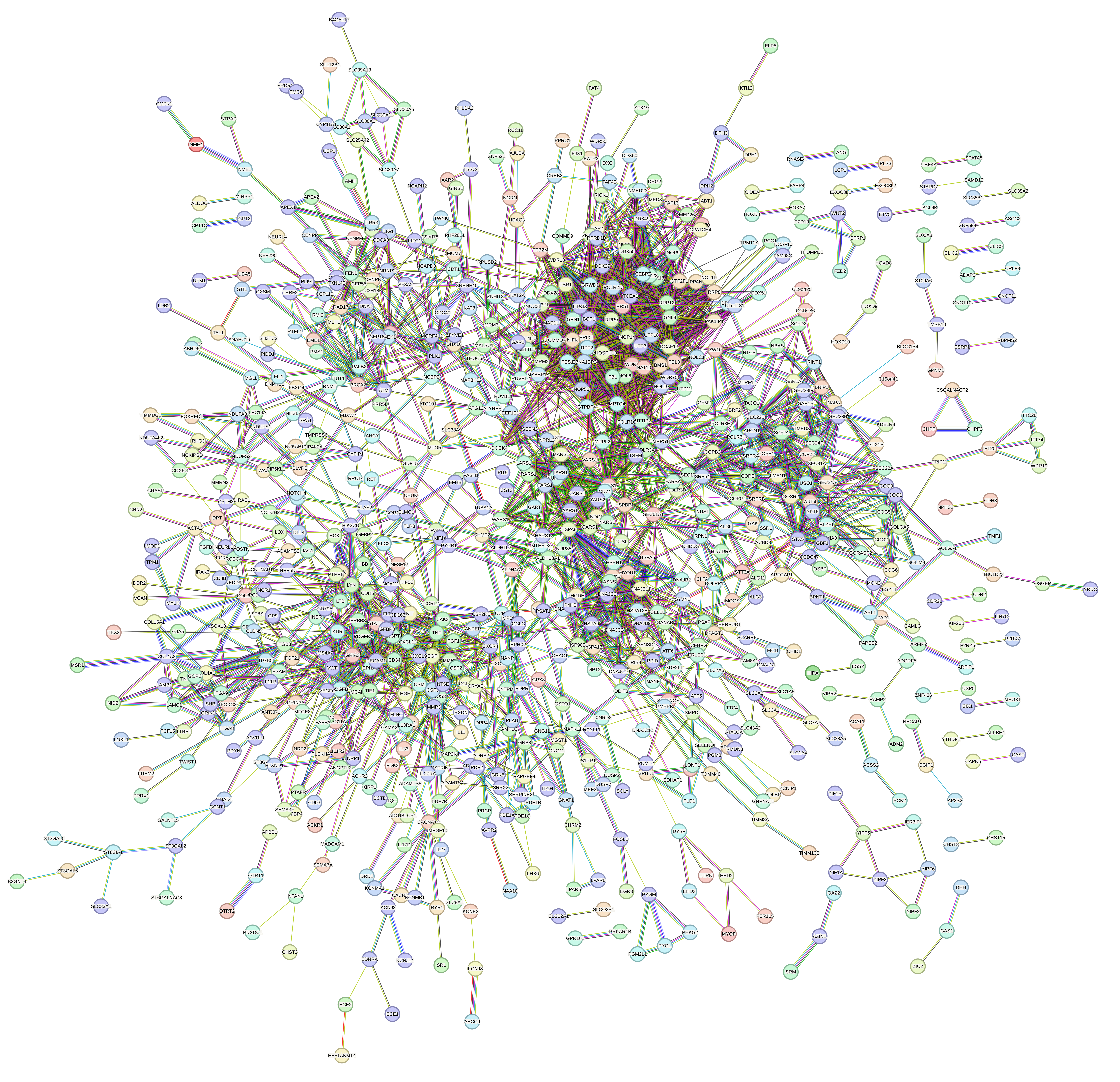


**Figure S3.** Predicted protein-protein interaction (PPI) network for all differentially expressed genes in endometrial explants of early pregnant gilts in response to UBCS039 treatment. The edges represent the predicted functional associations. There are seven types of evidence used in predicting the associations: database (light blue line), experimental (purple line), neighborhood (green line), gene fusion (red line), co-occurrence (blue line), text-mining (yellow line), and co-expression (black line) evidence.
