# Supplementary material for: Expression, regulation, and multifaceted molecular and biological functions of sirtuin 6 in the porcine endometrium during early pregnancy
Source: Cell Commun Signal. 2026 Feb 4;24:158. doi: 10.1186/s12964-026-02699-1 (PMC12958535; doi:10.1186/s12964-026-02699-1)
Supplement: Supplementary file 3 — Additional file 3: Figures S4-S8. Original images for Western blots. [file 12964_2026_2699_MOESM3_ESM.docx]

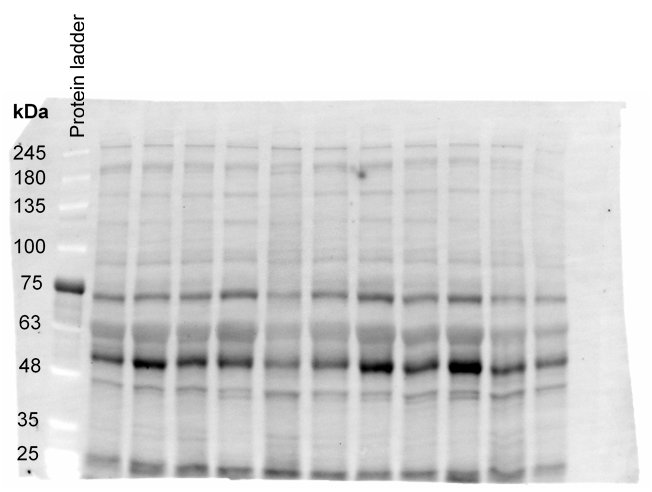
 **Blot #1 Stain-free blot #1**

**
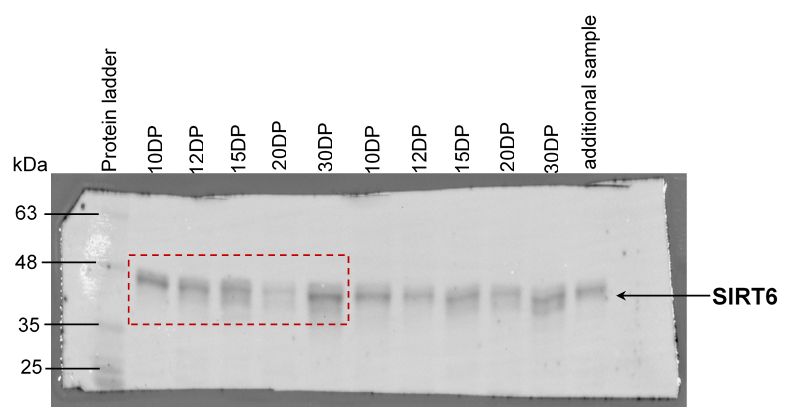
**


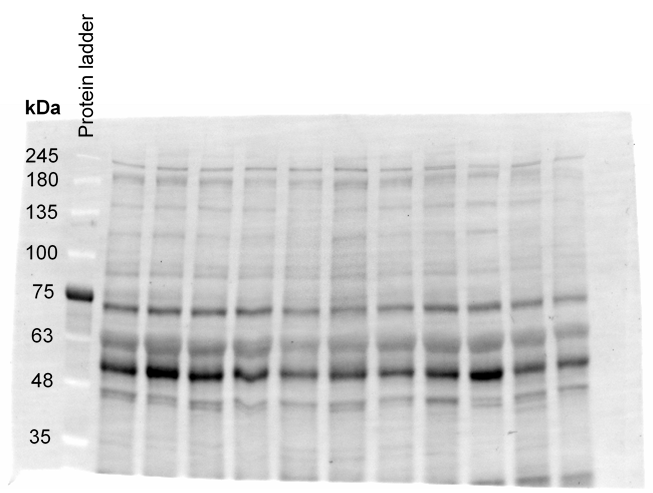
 **Blot #2 Stain-free blot #2**


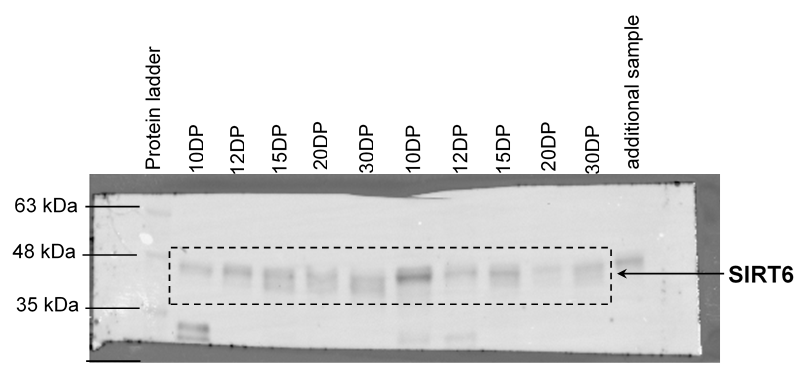


**
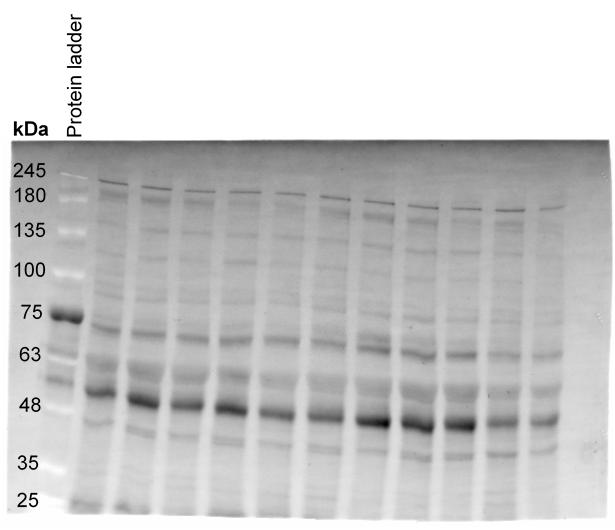
**

**
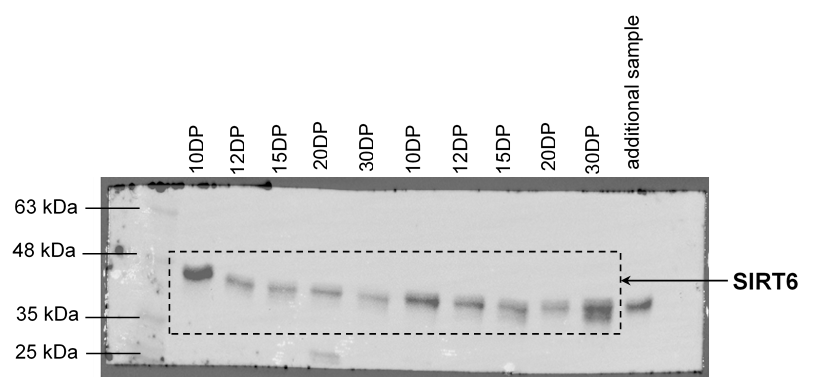
 Blot #3 Stain-free blot #3**

**Figure S4.** Western blot results showing sirtuin 6 (SIRT6) protein expression in endometrial tissue obtained from early pregnant gilts. The red dashed line shows fragments of the blot presented in Fig. 1b. Stain-Free blot images used for normalization of SIRT6 protein abundance relative to the total protein content in each equivalent lane are presented in the right panel. Blots were cut according to the molecular weight of the tested protein and based on the protein ladder. DP: day of pregnancy


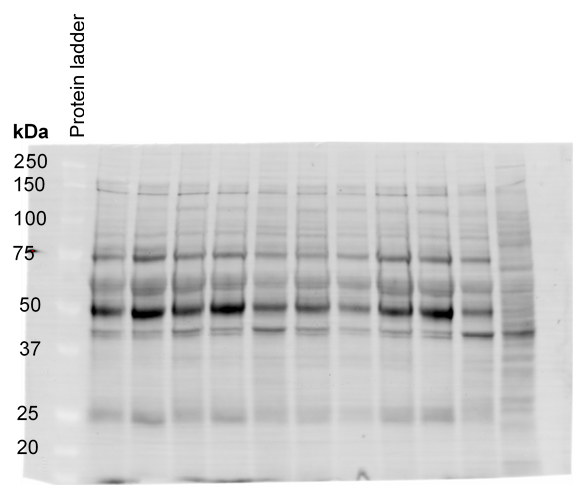

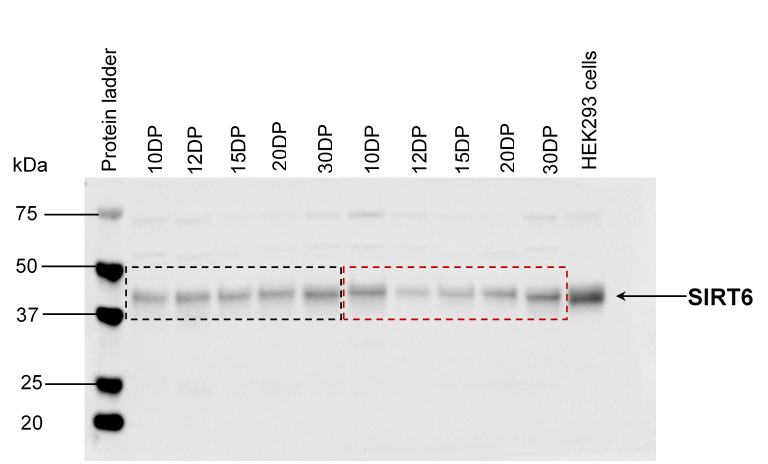
 **Blot #1 Stain-free blot #1**


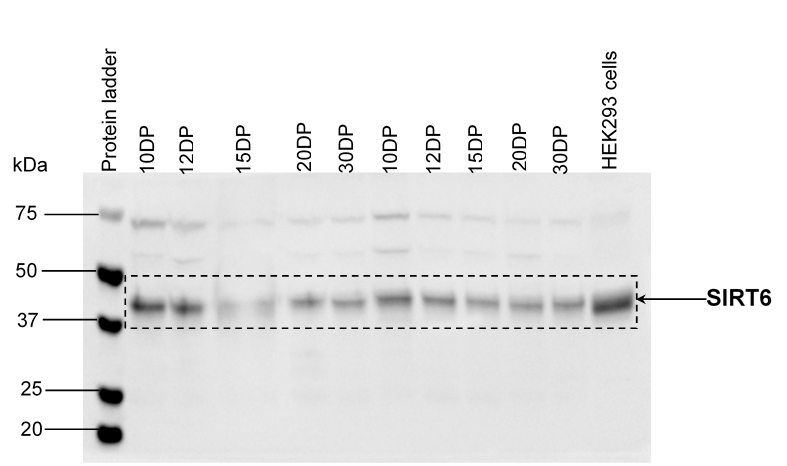

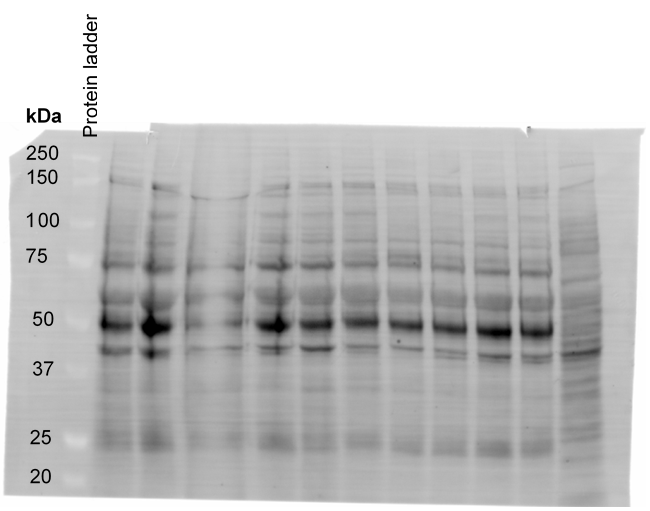


**Blot #2 Stain-free blot #2**

**
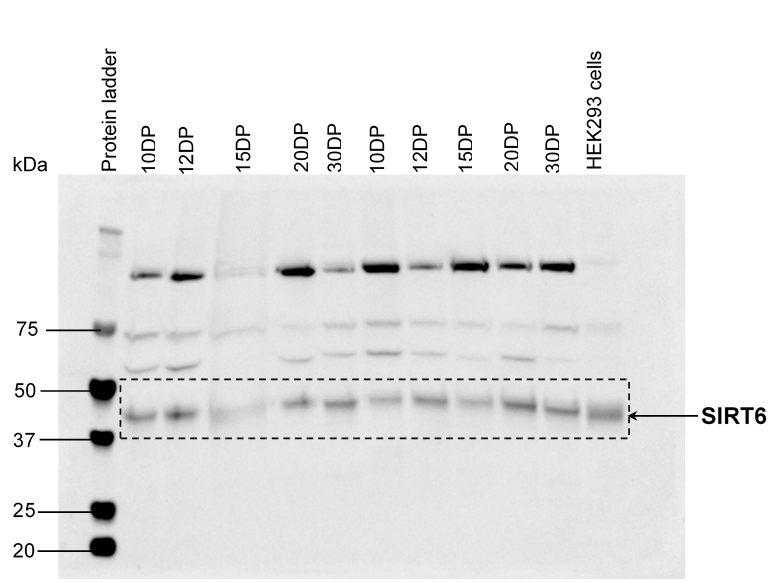
**

**
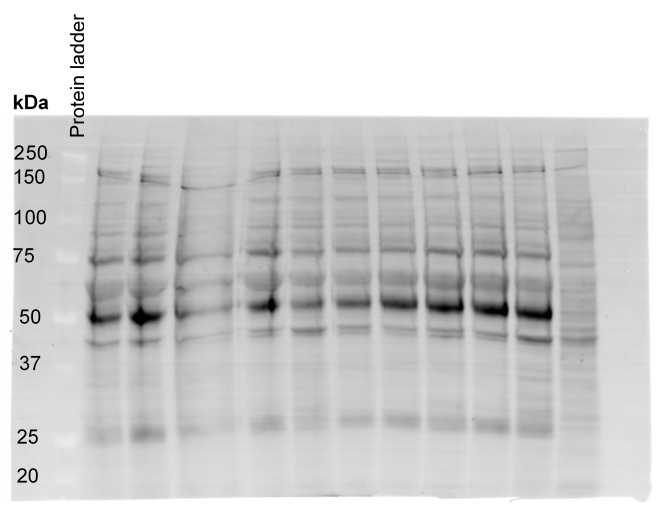
 Blot #3 Stain-free blot #3**

**Figure S5.** Western blot results showing sirtuin 6 (SIRT6) protein expression in nuclear fraction of endometrial tissue obtained from early pregnant gilts. The HEK293 cell line was used as a positive control. The red dashed line shows fragments of the blot presented in Fig. 1c. Stain-Free blot images used for normalization of SIRT6 protein abundance relative to the total protein content in each equivalent lane are presented in the right panel. Blots were cut according to the molecular weight of the tested protein and based on the protein ladder. DP: day of pregnancy.


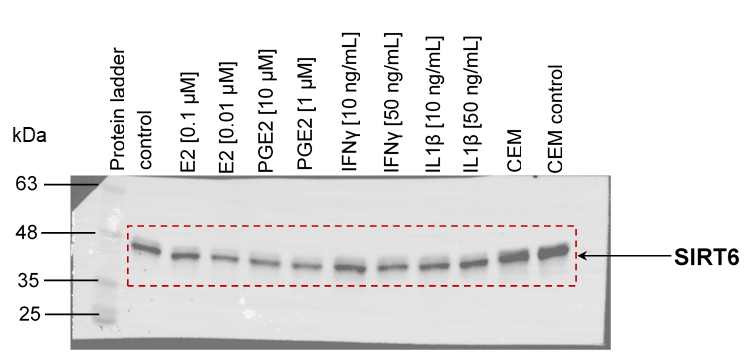

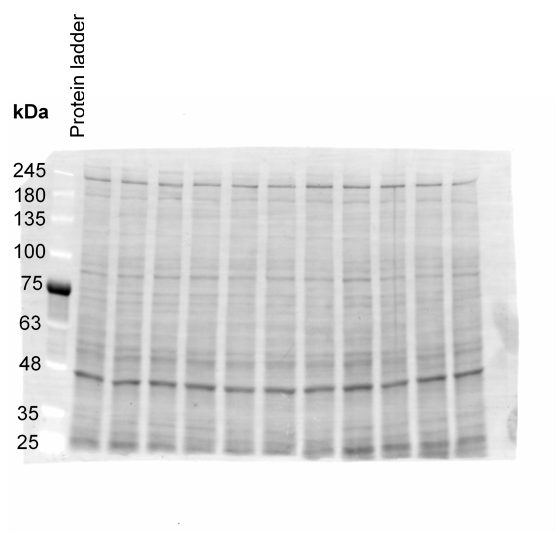
 **Blot #1 Stain-free blot #1**


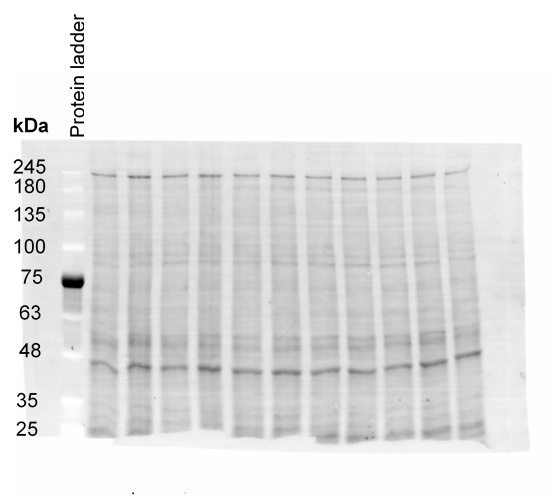


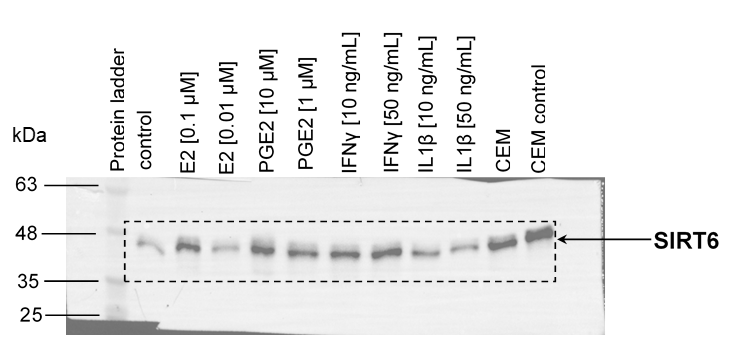
 **Blot #2 Stain-free blot #2**

**
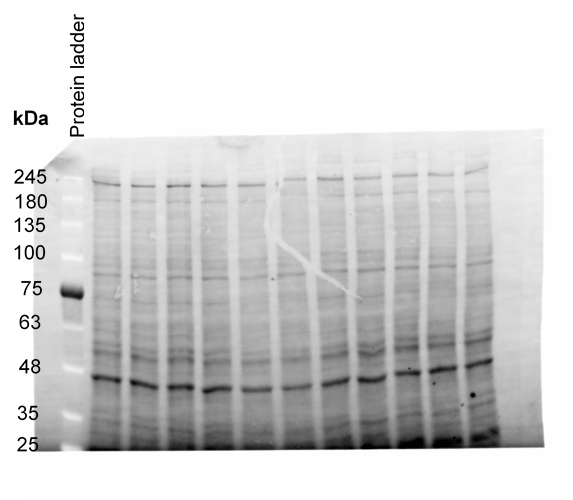
**

**
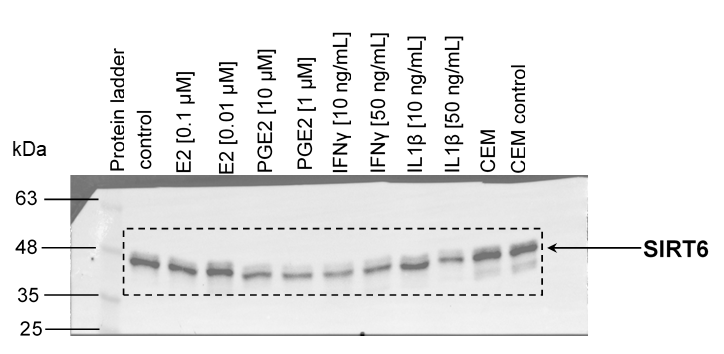
 Blot #3 Stain-free blot #3**

**
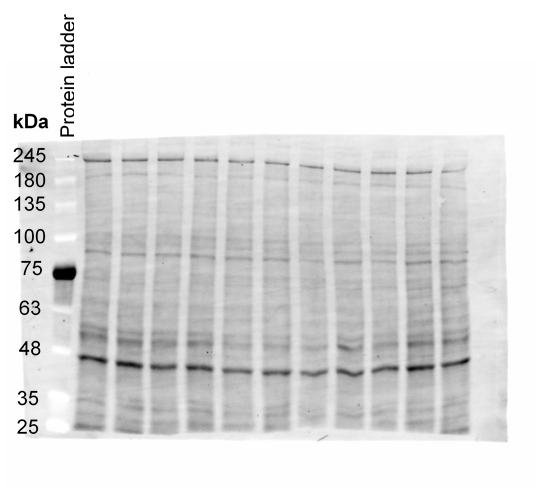
**

**
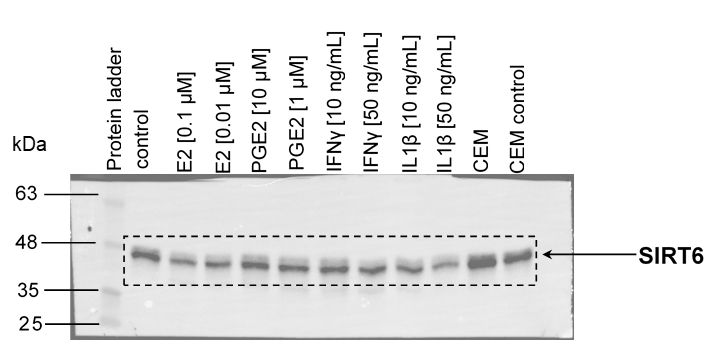
 Blot #4 Stain-free blot #4**

**
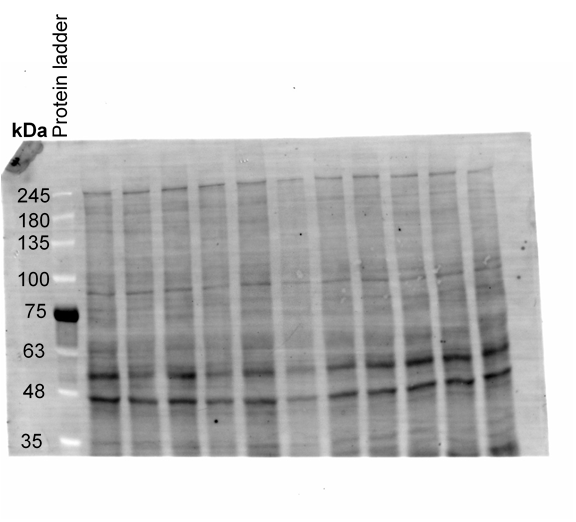
**

**
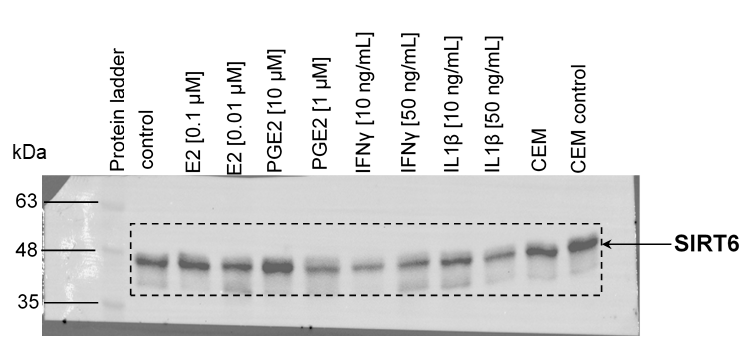
 Blot #5 Stain-free blot #5**

**Figure S6.** Western blot results showing sirtuin 6 (SIRT6) protein expression in endometrial slices treated with estradiol (E2; 0.01 and 0.1 µM), prostaglandin E2 (PGE2; 1 and 10 µM), interferon γ (IFNγ; 10 and 50 ng/mL), interleukin 1β (IL1β; 10 and 50 ng/mL), or conceptus-exposed medium (CEM) for 24 h. The red dashed line shows fragments of the blot presented in Fig. 1d. Stain-Free blot images used for normalization of SIRT6 protein abundance relative to the total protein content in each equivalent lane are presented in the right panel. Blots were cut according to the molecular weight of the tested protein and based on the protein ladder.

**
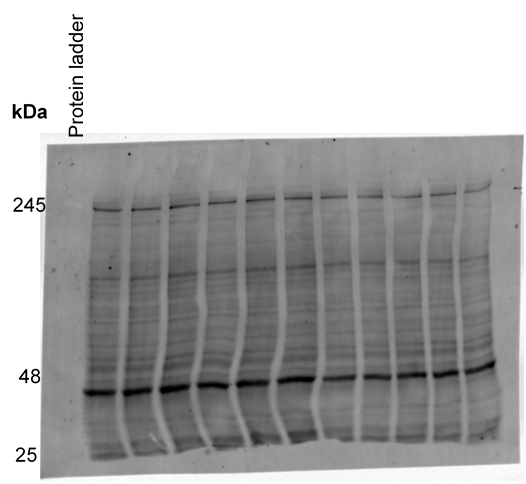

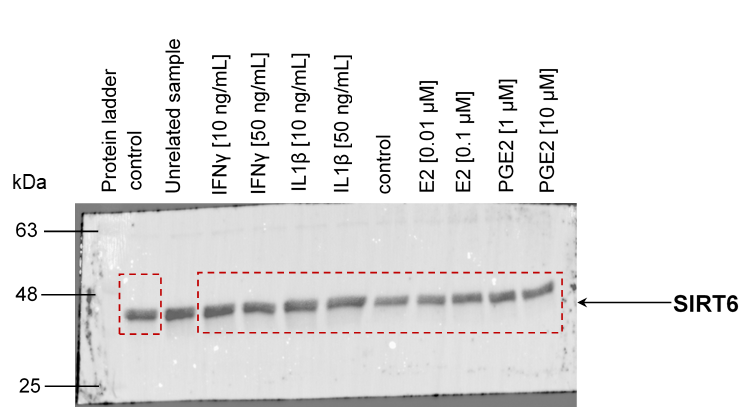
a) Blot #1 Stain-free blot #1**

**
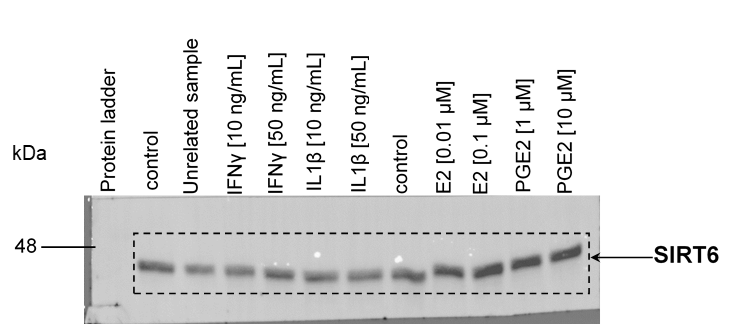

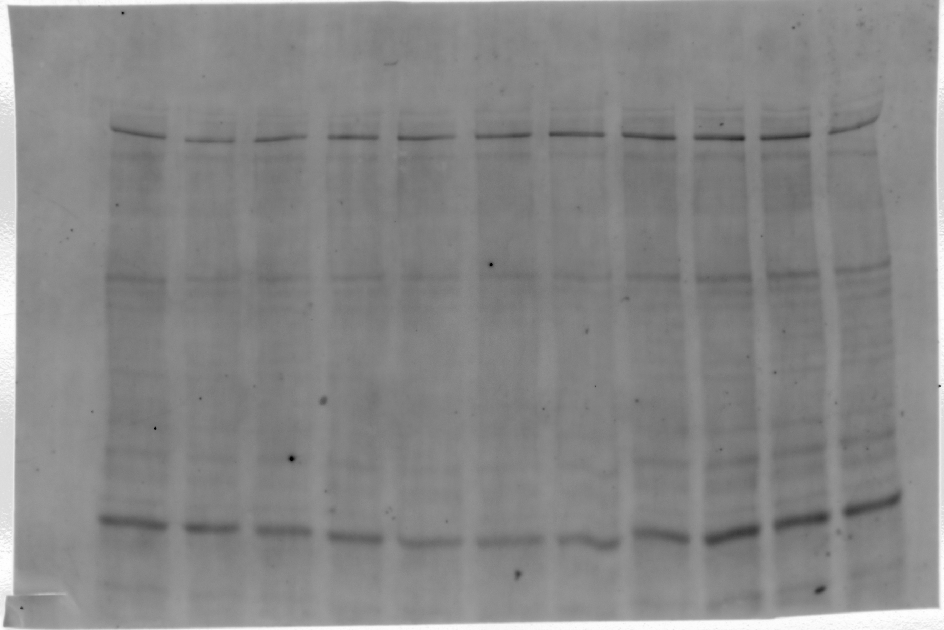
 Blot #2 Stain-free blot #2**

**
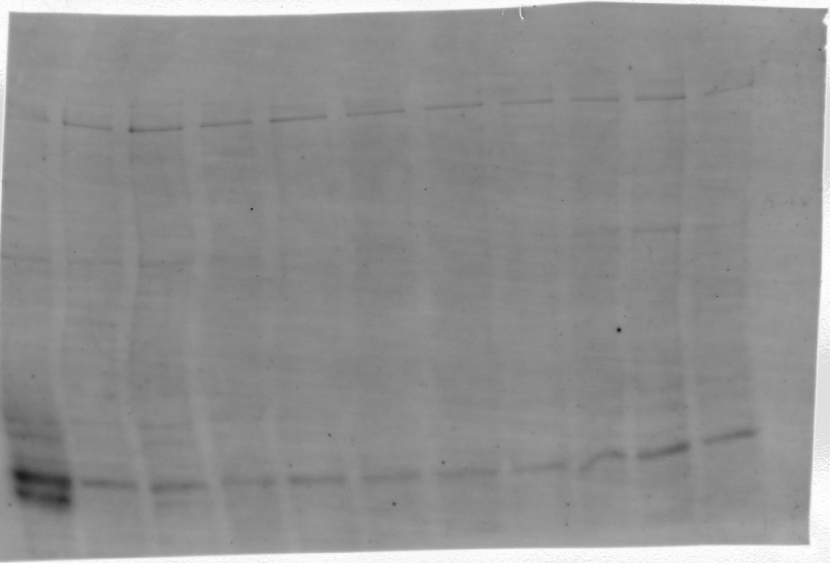

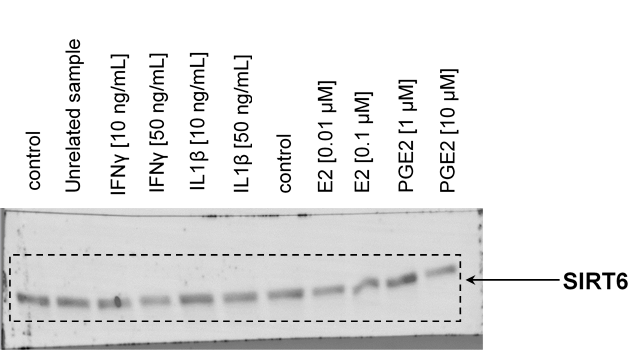
 Blot #3 Stain-free blot #3**

**
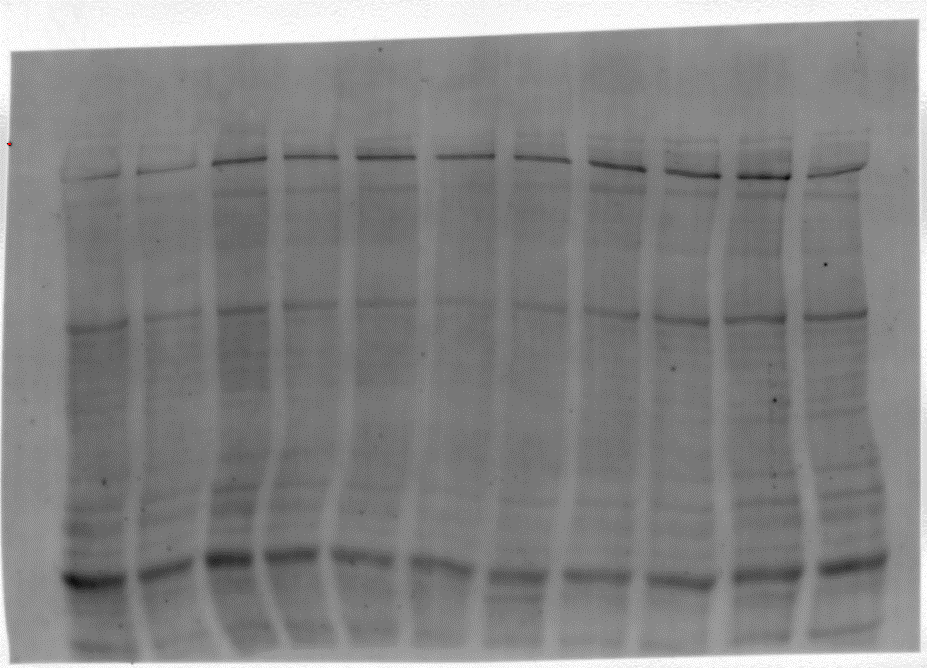

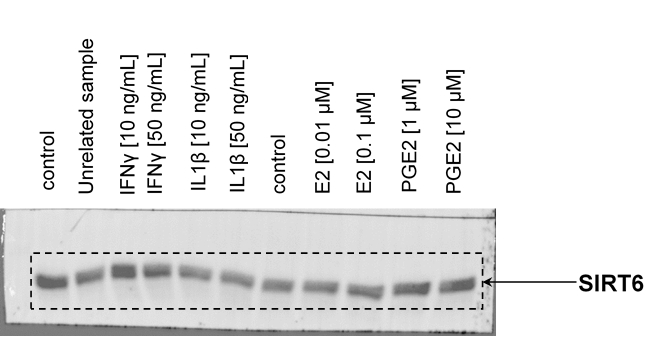
 Blot #4 Stain-free blot #4**

**
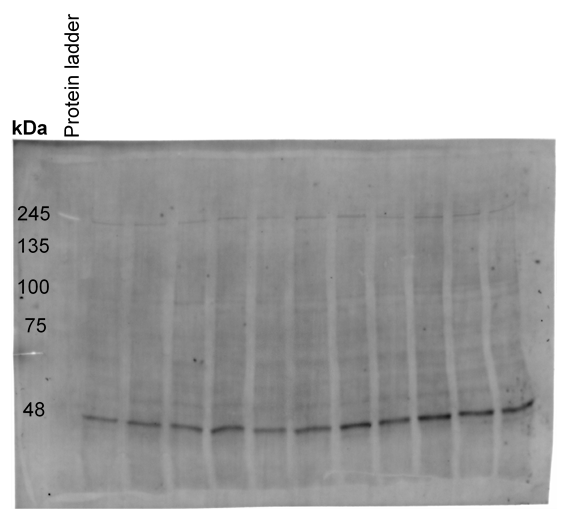
**

**
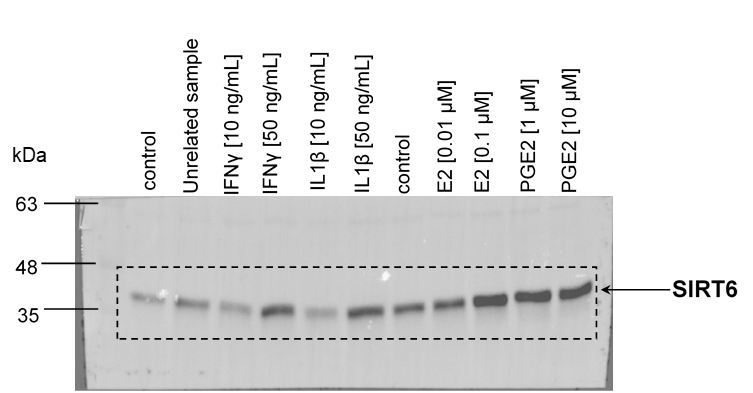
 Blot #5 Stain-free blot #5**

**
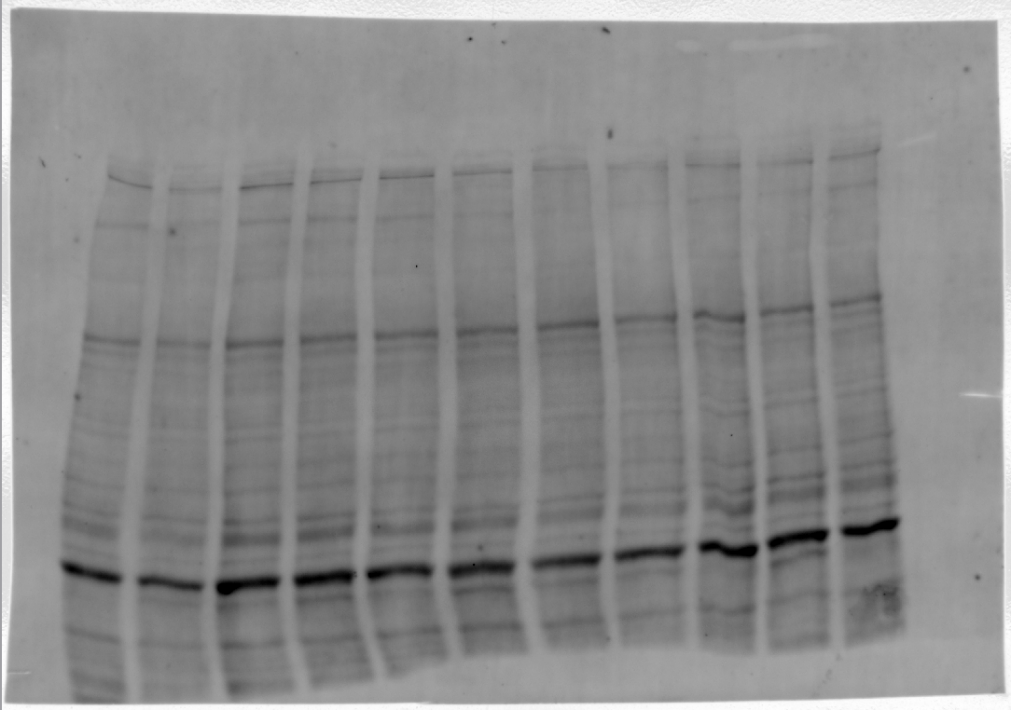

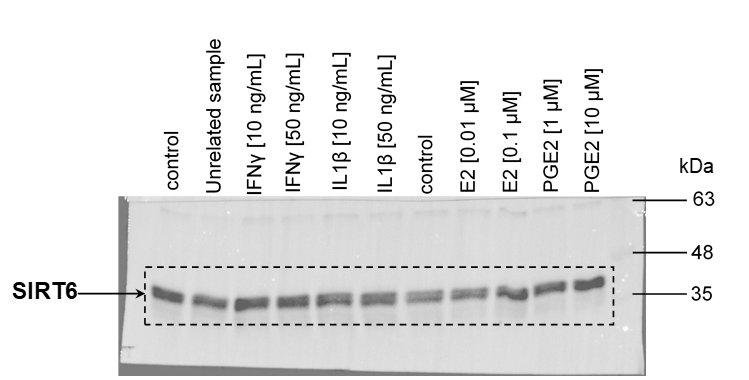
 Blot #6 Stain-free blot #6**

**
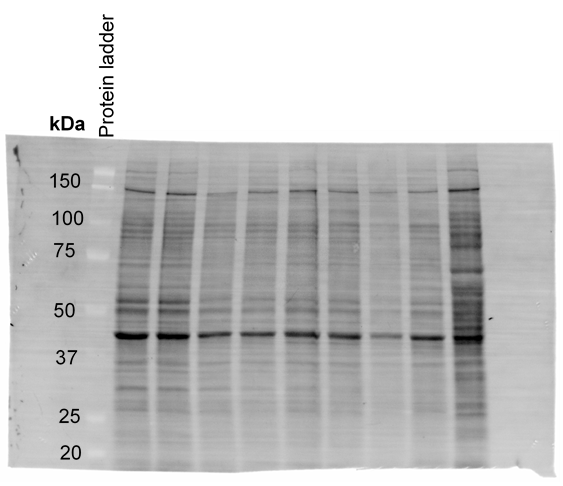

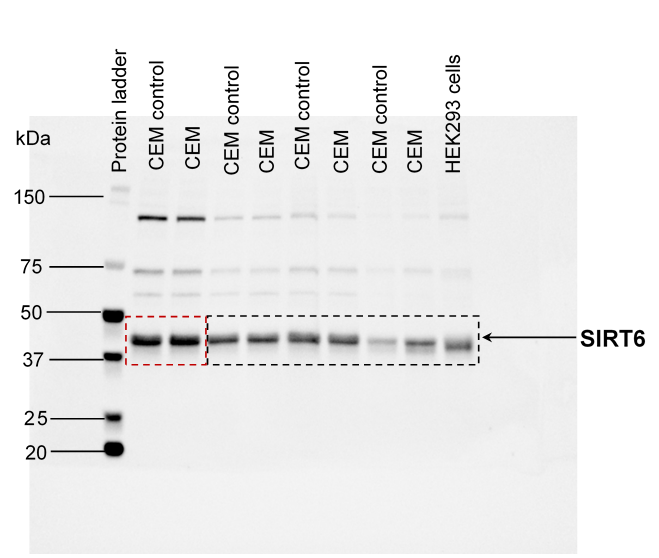
b) Blot #1 Stain-free blot #1**

**
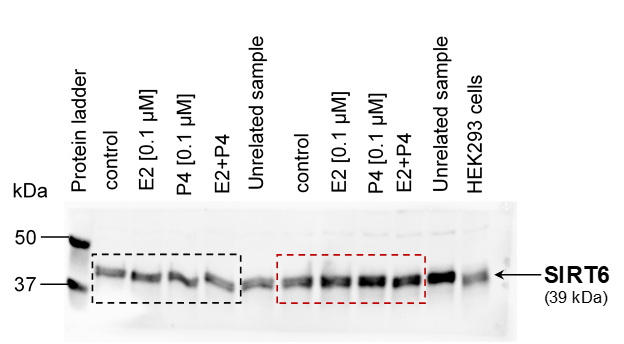
c) Blot #1**

**
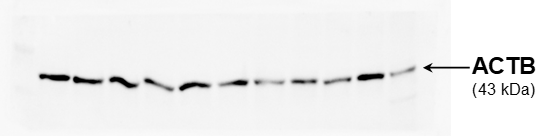
**

**
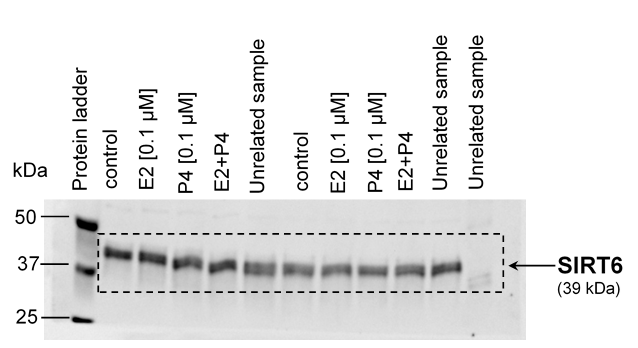
 Blot #2**

**
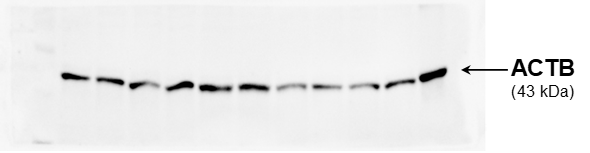
**

**
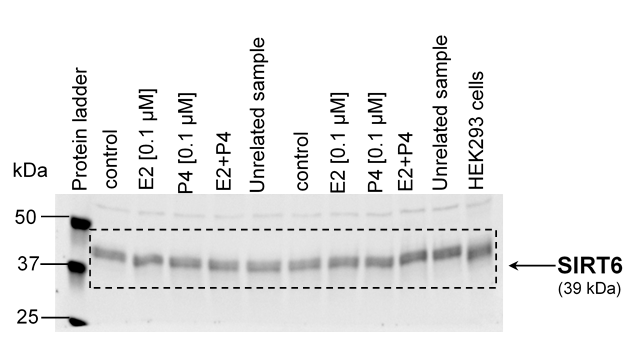
 Blot #3**

**
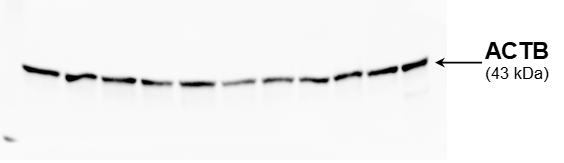
**

**Figure S7.** Western blot results showing sirtuin 6 (SIRT6) protein expression in luminal epithelial cells treated with **(a)** estradiol (E2; 0.01 and 0.1 µM), prostaglandin E2 (PGE2; 1 and 10 µM), interferon γ (IFNγ; 10 and 50 ng/mL), interleukin 1β (IL1β; 10 and 50 ng/mL), or **(b)** conceptus-exposed medium (CEM), or **(c)** E2 (10 µM), progesterone (P4; 0.1 µM), or the combination of E2 and P4 for 24 h. The HEK293 cell line was used as a positive control. The red dashed line shows fragments of the blot presented in Fig. 2. Right panel: (a, b) Stain-Free blot images used for normalization of SIRT6 protein abundance relative to the total protein content in each equivalent lane; (c) beta-actin (ACTB) used as an internal control of protein loading. Blots were cut according to the molecular weight of the tested protein and based on the protein ladder.


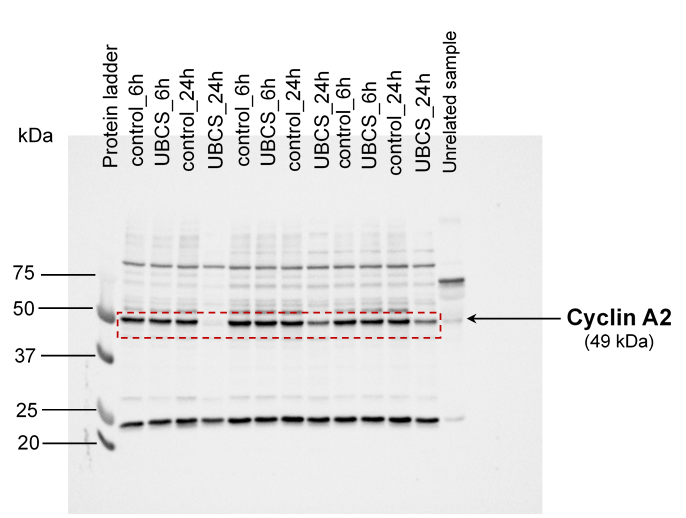


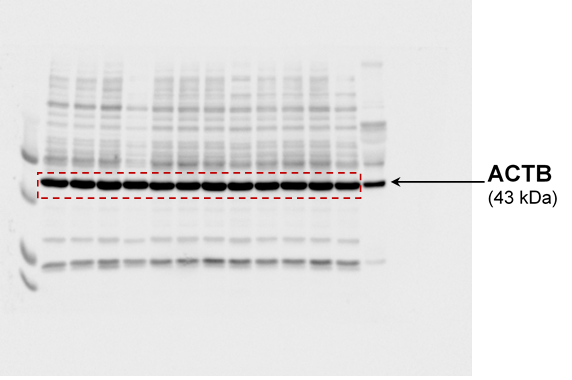


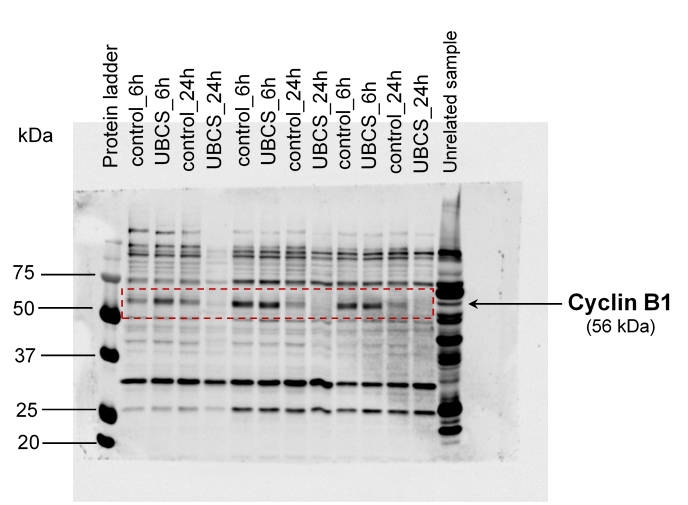


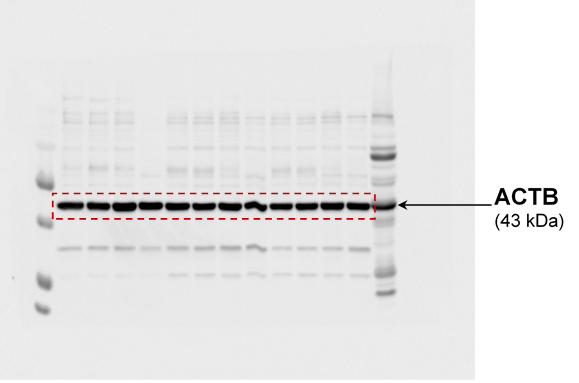


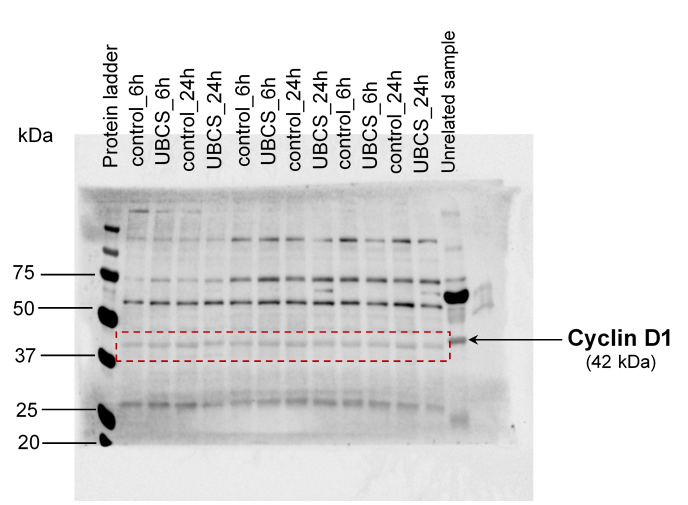


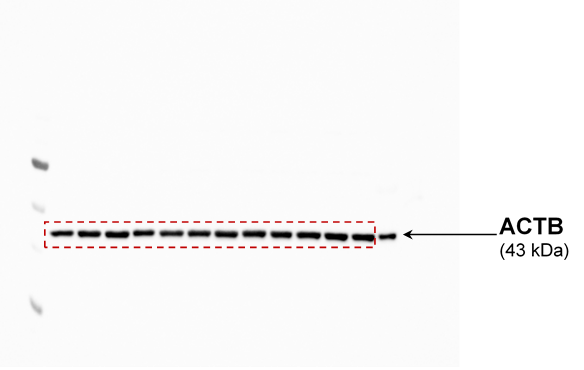


**
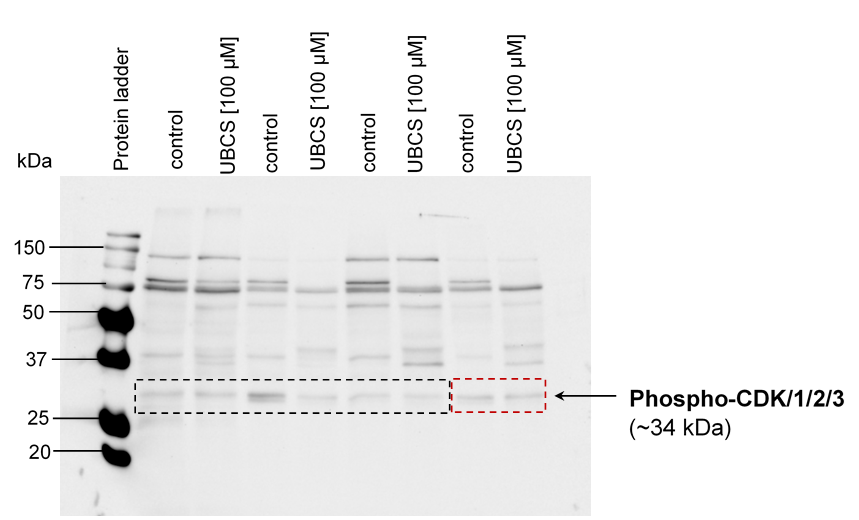
**

**
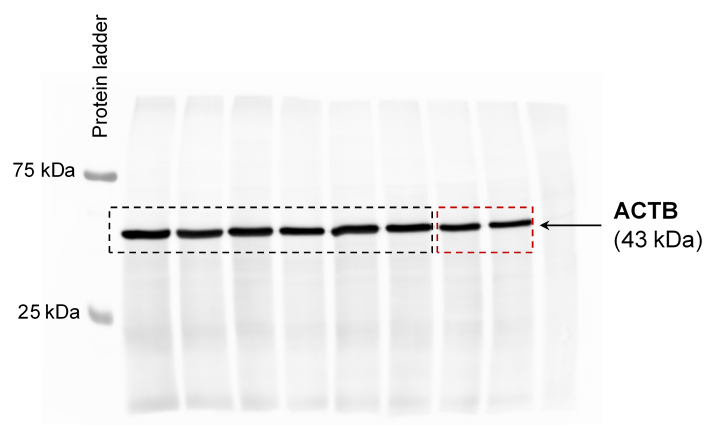
**

**Figure S8.** Western blot results showing the expression of cell cycle-related proteins in luminal epithelial cells treated with UBCS039 (100 µM) for 6 and/or 24 h. The red dashed lines show fragments of the blots presented in Fig. 7. Beta-actin (ACTB) was used as an internal control of protein loading (right panel).
